# Psychometric Properties of Instruments for Perpetration and Victimization of Dating Violence in Young People: Systematic Review and Meta-Analysis

**DOI:** 10.3390/ejihpe15040044

**Published:** 2025-03-24

**Authors:** Andrés Ramírez, Luis Burgos-Benavides, Hugo Sinchi-Sinchi, Javier Herrero Díez, Francisco Javier Rodríguez-Díaz

**Affiliations:** 1 Department of Clinical Psychology, Universidad Politécnica Salesiana, Cuenca 010107, Ecuador; 2 Department of Psychology, Universidad de Oviedo, 33003 Oviedo, Spain; burgosluis@uniovi.es (L.B.-B.); herrero@uniovi.es (J.H.D.); gallego@uniovi.es (F.J.R.-D.); 3 Department of Psychology, Pontificia Universidad Católica del Ecuador, Esmeraldas 080101, Ecuador; hfsinchi@pucese.edu.ec

**Keywords:** perpetration, victimization, adolescent, dating violence, reliability

## Abstract

**Introduction**: Adolescent dating violence is a public health issue with long-term implications for the emotional and psychological development of young people. Properly evaluating this issue requires instruments with solid psychometric properties. This study aims to identify and analyze the psychometric properties of the instruments used to measure perpetration and victimization in adolescent dating violence. **Objective**: Our objective was to identify the psychometric properties of the instruments measuring perpetration and victimization in adolescent dating violence through a systematic review of the scientific literature and meta-analysis of reliability and structural validity. **Methods**: The study design included a systematic review of the literature and a meta-analysis. The data sources used were scientific databases such as *PubMed*, *PsycINFO*, *Scopus*, and *Web of Science*. Inclusion criteria were studies that evaluated the reliability and validity of instruments measuring adolescent dating violence, published in English and Spanish. Data analysis was performed through a meta-analysis to calculate reliability indices, such as Cronbach’s alpha, and structural validity was assessed using factor analysis techniques. **Results**: The results showed high reliability and structural validity in the instruments measuring dating violence, with high global alpha and omega coefficients and excellent model fit indices. However, heterogeneity was significant, and lower coefficients in measuring sexual violence indicated areas for improvement. Limitations include variability between studies, possible publication biases, and a lack of detailed demographic data. **Conclusions**: The instruments are generally reliable and valid, but more research is needed to improve the accuracy in measuring sexual violence and to ensure generalization in diverse contexts.

## 1. Introduction

Adolescent dating violence (ADV) victimization and perpetration are significant issues with long-term adverse consequences, including internalization of symptoms, externalization of behaviors, substance use, and revictimization (Rodríguez-Franco et al., 2022; Campo-Tena et al., 2024; Pereda et al., 2018; Psychogiou et al., 2023; Wolfe et al., 2001). In 2021, studies indicated that high school students in the U.S. reported various forms of interpersonal violence (IPV) victimization, such as physical and sexual violence in adolescent dating (TDV), sexual violence, and harassment, with disparities observed among different demographic groups (Clayton et al., 2023). Moreover, research underscores the urgent need for interventions targeting the victimization and perpetration of partner violence among adolescents and young adults in sub-Saharan Africa, highlighting the importance of addressing correlations across different stages of development (Johnson et al., 2024). The intersection of economic abuse, transactional sex, and reproductive coercion in adolescent dating relationships further reveals the complexity and severity of these issues, necessitating awareness among providers and agencies that serve youth (Scott et al., 2023). Additionally, LGBTQ youth also face elevated risks, with physical victimization in dating violence associated with higher likelihoods of suicide attempts, emphasizing the need for inclusive prevention strategies (Price et al., 2023).

In this context, the dating phase in adolescence is crucial for the emotional and social development of young people. Research shows that during this period, adolescents experience significant changes in their social interactions, emotional well-being, and brain development (Wesche et al., 2023). Studies highlight the prevalence and impact of adolescent dating violence (ADV), emphasizing the need for comprehensive prevention strategies that address multiple levels of the socio-ecological model to mitigate negative mental health consequences (Stepanous et al., 2023). Additionally, exposure to intimate partner violence (IPV) during childhood can significantly influence adolescent dating behavior and the quality of their relationships, underscoring the importance of early detection and interventions to support victims and promote healthy relationships (Claussen et al., 2022). Furthermore, gender dynamics and power imbalances in early dating practices can shape emotional attachment and perceptions of vulnerability among heterosexual youth, influencing relationship progression (Cheung & Huang, 2022).

Similarly, research on adolescent dating violence reveals significant findings. Studies have shown that adolescent exposure to media, such as television and video games, is associated with dating violence victimization (Rostad et al., 2021). Furthermore, experiences of child sex trafficking in the domestic sphere are linked to community violence victimization among adolescents, highlighting the interconnected nature of these forms of violence (Franchino-Olsen et al., 2022). Internalizing symptoms in adolescents, such as depression and anxiety, is simultaneously associated with both victimization and the perpetration of dating violence, emphasizing the bidirectional relationship between mental health and dating violence (Litz & Holvoet, 2021). Additionally, exposure to violent video games has been linked to an increased risk of perpetration and victimization of physical bullying among Chinese adolescents, indicating a specific association between exposure to violent games and different subtypes of bullying involvement (She et al., 2022). Together, these studies underline the complex interaction between various forms of violence and victimization experienced by adolescents in different contexts.

To address these issues, research on adolescent dating violence victimization and perpetration often employs various instruments to assess these behaviors. The Adolescent Conflict in Relationships Inventory (CADRI) is commonly used to measure different forms of conflict, including physical, emotional, and verbal abuse (Johnson et al., 2024). Additionally, the Conflict Tactics Scale (CTS2S) is used to assess various conflict resolution strategies, including violent behaviors such as physical and psychological aggression (Marr et al., 2024). The Juvenile Victimization Questionnaire (JVQ) (Almeida et al., 2020) evaluates a wide range of victimization experiences among adolescents, providing a comprehensive understanding of the prevalence and impact of different forms of victimization (Gámez-Guadix et al., 2023). These instruments are crucial for capturing the complexities of perpetration and victimization in adolescent dating violence and help researchers develop specific interventions and prevention strategies.

On the other hand, new instruments such as the CyDAV-T have been developed to differentiate between the sexual, verbal, and control dimensions of cyber violence in dating (Sánchez-Jiménez et al., 2023). Additionally, studies have used instruments like CADRI (Benítez & Muñoz, 2014), CTS2S (Muñoz-Rivas et al., 2007), and JVQ (Almeida et al., 2020) to assess patterns of victimization and perpetration of interpersonal violence among adolescents, identifying classes of violence experiences and their associations with demographic and behavioral health variables (Sessarego et al., 2021).

Studies have also explored the prevalence of electronic intrusion as a form of cyber abuse in dating relationships and its relationship with in-person violence, underscoring the need to incorporate electronic intrusion into theoretical models of dating violence (Doucette et al., 2021). Furthermore, meta-analytic research has shown significant relationships between bullying perpetration and dating violence, as well as between bullying victimization and dating violence victimization, suggesting the importance of personalized interventions to address these interconnected behavioral issues (Mozley et al., 2021). Contemporary approaches to evaluating the adequacy of psychometric instruments focus on a broad range of considerations in addition to internal reliability and structural validity—see, for example, the approach adopted by Meinck et al. (2023), who conducted a COSMIN systematic review to assess the psychometric properties of child and adolescent self-report measures of violence. Their findings highlight the need for comprehensive evaluations that consider measurement invariance, criterion validity, and responsiveness to change, ensuring that instruments are robust and applicable across diverse populations (Meinck et al., 2023).

The general objective of this study was to identify the psychometric properties of instruments used to assess perpetration and victimization of adolescent dating violence through a systematic literature review and meta-analysis of reliability and structural validity. The specific objectives were threefold: The first objective was to identify the key characteristics of publications focused on assessing perpetration and victimization of adolescent dating violence, based on parameters such as year of publication, journal name, the instrument used, sample size, number of scale items, reported internal reliability (Cronbach’s alpha and McDonald’s omega), and any fit indices related to the underlying factorial structure (CFI, TLI, RMSEA, and SRMR). Second, joint estimates of reliability and fit indices documented for measures of adolescent dating violence perpetration and victimization were calculated across all studies. Finally, heterogeneity between studies was assessed based on the reported metrics to discern variability across different assessment tools. This research will provide a deeper understanding of the psychometric state of the field of adolescent dating violence perpetration and victimization assessment while uncovering potential areas that require further examination to strengthen the methodological foundations in this area.

## 2. Materials and Methods

### 2.1. Study Design

This study followed the Preferred Reporting Items for Systematic Reviews (PRISMA, Page et al., 2021) and Meta-analysis of Reliability (López-Ibáñez et al., 2024) guidelines.

### 2.2. Inclusion and Exclusion Criteria

For this study, prevalence studies as well as longitudinal and cross-sectional studies were included. Participants had to be adolescents in a dating relationship. It was essential that the evaluated studies measured perpetration and/or victimization of adolescent dating violence. Only studies that used validated instruments to measure this violence were considered. The studies were required to report data on the prevalence of adolescent dating violence and/or the reliability (Cronbach’s alpha and McDonald’s omega) and fit indices (CFI, TLI, RMSEA, and SRMR) of the measurement instruments used. Additionally, studies published in English or Spanish and peer-reviewed in academic journals were included.

Studies focusing on partner relationships, such as violence in marriage, were excluded. Studies addressing other unrelated types of violence, such as domestic violence or bullying, were also excluded. Studies that used measurement instruments not validated through psychometric properties of reliability and validity were not considered. Studies that did not provide specific data on the prevalence of dating violence or the reliability of the measurement instruments were excluded. Systematic reviews and meta-analyses, as well as non-peer-reviewed works such as theses, dissertations, technical reports, or unpublished documents, were excluded. The authors state that the bibliographies of the articles included in the review were also examined to reduce exclusion bias due to coverage. In line with this, relevant studies were identified not only through database searches but also through reference screening. To enhance transparency and methodological rigor, we recommend using the PRISMA template designed for data obtained from multiple sources.

### 2.3. Sources of Information

Four scientific databases were used as information sources: *Web of Science*, *Scopus*, *PsycINFO*, and *PubMed*, with access provided by the University of Oviedo (Spain) and the Universidad Politécnica Salesiana (Ecuador). These databases were selected for their international scientific relevance and their high-quality coverage of studies in the social and health sciences. Additionally, the bibliographies of the included articles were reviewed to minimize exclusion bias due to coverage limitations. To ensure a comprehensive and standardized search strategy, the selection of keywords was conducted using the Descriptors in Health Sciences (*DeCS*) and Medical Subject Headings (*MeSH*), optimizing consistency in indexing across databases. The search terms included concepts related to intimate partner violence (e.g., Partner Violence Intimate, Intimate Partner Abuse, Dating Violence, Gender-Based Violence, Violence Against Women), target populations (e.g., Adolescents, Teenagers, Youth, Female Adolescents, Male Adolescents), and psychometric evaluation (e.g., Adaptation, Validation, Invariance, Psychometric, Validation Studies, Validation Scale, Instrument). The complete search phrases, applied filters, and Boolean operators used in each database are detailed in Supplementary Table S1.

### 2.4. Search Strategy

Searches in the four mentioned databases were conducted on 17 June 2024, following the guidelines outlined in the PRISMA statement (Page et al., 2021). The initial screening identified duplicate records, both algorithmically and through manual verification, with over 90% content overlap. Secondly, records focusing on subjects unrelated to dating violence were excluded. Systematic reviews, theoretical documents, and non-systematic narrative reviews were considered ineligible at this stage.

**Figure 1:**
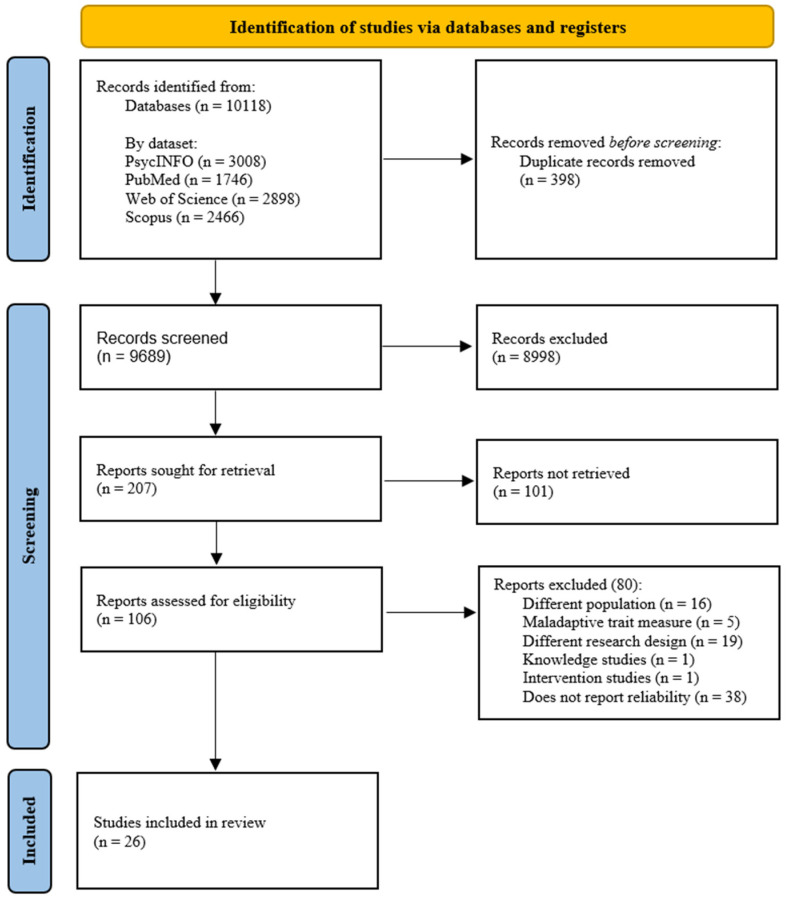
Flow diagram for systematic review and meta-analysis of the instruments of perpetration and victimization of dating violence (*n* = 10,118). From: Page et al. (2021).

Two independent researchers reviewed the full text of the remaining records, comparing them with the pre-established inclusion criteria. Disagreements regarding coding decisions led to deliberation until consensus was reached, and the judgments were classified as included, excluded, or requiring further discussion. Coding for this phase was facilitated using the web application Rayyan (Ouzzani et al., 2016), with classifications as duplicate, deleted, included, excluded, and possibly included. The underlying reasons for these decisions were recorded in the system. Finally, blinding was disabled, allowing the researchers to cross-check results and proceed with the study (Figure 1).

### 2.5. Data Collection Process

A thorough process of systematic bibliographic data extraction was carried out for all records considered eligible from the selected databases. The collected data included author names, publication titles, source journals, and abstracts, providing a comprehensive overview of each record. One researcher downloaded these data in RIS format, a standard for bibliographic data exchange, managing each record individually by database. These RIS files were then verified and uploaded into Rayyan (Ouzzani et al., 2016), a specialized tool for managing and reviewing scientific literature. To ensure accuracy and reduce biases, an independent researcher was included as a blind coding partner, allowing for a dual screening process. This meticulous protocol of extraction and processing was followed sequentially across the four databases, ensuring consistency and rigor in handling the information.

### 2.6. Data Extraction Process

For data extraction, two categories were defined: (1) the methodological characteristics of the included studies, and (2) the psychometric properties of the instrument. The lead reviewer identified the information according to the variables defined in the work matrix and cross-checked it with the external reviewer. Any inconsistencies were resolved by comparing and verifying the data together until agreement was reached.

Regarding the methodological variables, the following data of interest were collected: (1) author and year of publication; (2) study title in the original language of publication; (3) journal and quartile; (4) psychometric instrument and number of items in the structure; (5) country, sample size, and average age of participants; (6) reliability measures and fit indices of the instruments used for assessing perpetration and victimization of adolescent dating violence.

The psychometric data of interest were: (1) reliability as measured by Cronbach’s alpha and McDonald’s omega; (2) goodness of fit of the hypothetical structural model defined by the Comparative Fit Index (CFI); (3) divergence between hypothetical and data-based models, measured by the Root Mean Square Error of Approximation (RMSEA); (4) the Tucker–Lewis Index (TLI); (5) the Standardized Root Mean Square Residual (SRMR).

### 2.7. Evaluation of the Risk of Bias of the Study

The lead reviewer (AR) and the second reviewer (HS) analyzed the methodological quality of the included studies by reviewing the reliability or internal consistency based on Cronbach’s alpha of the psychometric instruments used to assess alcohol consumption. A Cronbach’s alpha value of 0.70 was considered an acceptable threshold, in line with the adopted standards. The representativeness of the sample was evaluated in relation to the number of items in the instrument reported by each study. The reviewers agreed on 96.96%, and discrepancies were resolved through deliberation and consensus with a third reviewer (LEB).

Regarding the 26 studies that reported the statistical data of interest, the researchers (HFS; AR) decided to document the samples separately by reviewing the full text and then cross-checking the data to reach consensus through deliberations.

The risk of bias analysis was thoroughly reviewed, and all potential biases were fully addressed. Specifically, the analysis focused on the consistency of psychometric instruments and the adequacy of the sample size in relation to the instrument’s design. In addition, other sources of bias, such as selection bias, reporting bias, and biases due to measurement errors, were considered to ensure the analysis was as exhaustive as possible. As a result, the study’s risk of bias assessment provides a comprehensive view of the factors that could influence the reliability and validity of the findings, thereby strengthening the overall robustness of the conclusions drawn from the included studies.

### 2.8. Statistical Analysis

This investigation employed reliability generalization and meta-analytic approaches to measure the internal consistency and model fit metrics of assessment instruments for Dating Violence across published studies. Cumulative reliability was estimated utilizing Cronbach’s alpha values transformed using the Hakstian–Whalen method to facilitate random-effects modeling (Hakstian & Whalen, 1976). Qualified studies also provided model fit data, including measures such as the comparative fit index (CFI), Tucker–Lewis index (TLI), root mean square error of approximation (RMSEA), and standardized root mean square residual (SRMR). Heterogeneity was assessed through Cochran’s Q, *I*^2^, H^2^, and τ^2^ metrics (Higgins & Thompson, 2002). To ensure result reliability, studies deviating from criteria such as alpha magnitudes > 0.700 or a minimum 10:1 cases-per-item ratio were removed using trimming techniques, as well as those with inadequate cases-per-item ratios before calculating pooled estimates. Potential publication bias was evaluated through visual inspection of funnel plot asymmetry and Egger’s regression test (L. Lin et al., 2018). Through meta-analysis of reliability and validity indicators in various applications, this study facilitates generalization regarding the efficacy of existing tools in the consistent measurement of dating violence across diverse samples. All analyses were conducted using Jamovi version 2.6.26, JASP version 0.19.3, R version 4.4.3 y Stata version 18.

## 3. Results

Of the 27 studies, categorized according to various criteria such as authors, year of publication, and journal in which they were published, the following observations can be made: In terms of authors, each one represents 3.7% of the total, except for Fernández-González et al. (2012), who appear twice, constituting 7.4% of the total. This indicates that most of the studies were conducted by different researchers, suggesting a diversity of sources in data collection.

Regarding the year of publication, the studies are distributed between 2006 and 2024. The year 2021 stands out with 8 studies, representing 30% of the total. Other years with more than one publication include 2015 and 2018, each with 3 studies (11%), and 2006 and 2012, each with 2 studies (7.4%). This could indicate a rise in interest or an increase in relevant research production in 2021.

Concerning the journals, publications are spread across several of them. The journals International Journal of Clinical and Health Psychology and Violence and Victims have the highest number of publications, with 3 studies each (11%). Other journals with more than one publication include Advances in Mental Health, International Journal of Environmental Research and Public Health, and Journal of Interpersonal Violence, each with 2 studies (7.4%). The variety of journals suggests that the studies cover a wide range of approaches and areas of interest within the fields of psychology and public health.

In 2006 and 2012, 2 studies were published each year (7.4%). In 2007, 2010, 2014, 2016, 2017, 2019, 2022, 2023, and 2024, each of these years saw a single study published (3.7%). The year 2015 showed a slight increase with 3 studies (11%). Another year with significant activity was 2018, also with 3 studies (11%). The year that stands out the most is 2021, with 8 studies published (30%). This suggests a notable peak in the amount of research published in that year compared to others.

On the geographic front, the data reveal that Spain is the country with the highest representation in the studies, contributing 41%. This indicates significant interest and a high level of research production in this area within Spain. Following are Canada and the United States, contributing 15% and 11%, respectively, while other countries such as Bolivia, Chile, China, Mexico, Peru, and Turkey have much smaller representation, each with 3.7% of the studies. This geographic distribution highlights the variability in academic attention and resources devoted to studying dating violence in different regions (Table 1).

Regarding the instruments used to measure dating violence, there is a notable diversity. The most commonly used instrument is the CADRI, employed in 15% of the studies, followed by the DVQ at 11%. Other instruments, such as the CADRI-S, ADV–YL, CTS 2, and several others, are used in 3.7% of the studies each. This variety of tools reflects the broad scope of the field and the lack of a unified consensus on the best methodology to assess dating violence. Each instrument likely addresses different aspects or dimensions of the phenomenon, which can enrich the overall understanding but may also introduce challenges in comparing results across studies.

Finally, Table 2 shows that most instruments identify two factors of dating violence, representing 67% of the studies. This suggests a trend toward simplifying the conceptualization of dating violence, likely to facilitate the interpretation and application of results. However, some studies identify up to eight factors, indicating more detailed and complex approaches. These more detailed studies may provide a richer and more nuanced understanding of the phenomenon, although they may also be more difficult to generalize and apply in broader contexts. Taken together, these data offer a comprehensive view of research on dating violence, highlighting both common trends and areas of diversity in the methodological approach.

Table 3 presents the summary measures of the sample, items, reliability, and fit measures of the analyzed studies. Regarding the mean age, 26 studies reported a mean of 18.74 years (SD = 2.19, Minimum = 15.89, and Maximum = 22.72). The minimum age reported in these studies is 15.34 years (SD = 2.46, Minimum = 11, and Maximum = 19). The maximum age reported has a mean of 22.84 years (SD = 3.47, Minimum = 18, and Maximum = 30).

The total sample comprises 27 studies, with a mean sample size of 1425.48 participants and a standard deviation of 2020.92, with sample sizes ranging from 100 to 8105 participants. The mean number of items per study is 25.51, with a standard deviation of 13.22, a minimum of 10, and a maximum of 59 items.

Regarding the reliability of the measures, Cronbach’s alpha (α), based on 25 studies, has a mean of 0.84 with a standard deviation of 0.07, and values ranging from 0.70 to 0.96. For perpetration, based on 7 studies, the mean alpha is 0.85 with a standard deviation of 0.05, with a range from 0.76 to 0.93. In victimization, based on 7 studies, the mean Cronbach’s alpha is 0.85 with a standard deviation of 0.04, and values ranging from 0.78 to 0.91.

Breaking down psychological perpetration, based on 8 studies, the mean Cronbach’s alpha is 0.78 with a standard deviation of 0.07, and values ranging from 0.64 to 0.87. For physical perpetration, in 7 studies, the mean alpha is 0.80 with a standard deviation of 0.05, with values ranging from 0.73 to 0.88. In terms of sexual perpetration, based on 6 studies, the mean alpha is 0.64 with a standard deviation of 0.19, with a range of 0.42 to 0.88.

For psychological victimization, in 8 studies, the mean Cronbach’s alpha is 0.77 with a standard deviation of 0.06, and values ranging from 0.64 to 0.83. In physical victimization, based on 7 studies, the mean alpha is 0.81 with a standard deviation of 0.06, with a range from 0.74 to 0.92. Finally, for sexual victimization, based on 6 studies, the mean alpha is 0.63 with a standard deviation of 0.18, with values ranging from 0.41 to 0.86.

Regarding other reliability measures of McDonald’s Omega (ω), based on 4 studies, the mean is 0.86 with a standard deviation of 0.09, with values ranging from 0.76 to 0.96. The CFI (Comparative Fit Index), based on 20 studies, has a mean of 0.95 with a standard deviation of 0.04, and values ranging from 0.80 to 1.00. The TLI (Tucker–Lewis Index), based on 10 studies, shows a mean of 0.95 with a standard deviation of 0.02, with values ranging from 0.91 to 0.99. The RMSEA (Root Mean Square Error of Approximation), based on 20 studies, has a mean of 0.04 with a standard deviation of 0.02, and values ranging from 0.00 to 0.07. Finally, the SRMR (Standardized Root Mean Square Residual), based on 5 studies, has a mean of 0.04 with a standard deviation of 0.02, with values ranging from 0.01 to 0.07.

The study analyzed the reliability and structural validity of instruments measuring dating violence perpetration and victimization using a random effects model and heterogeneity statistics (Table 4). The results for the global alpha coefficient showed a high level of reliability with an estimate of 0.82 and a standard error (SE) of 0.0143. The Z value was 58.8, and the *p*-value was less than 0.001, indicating strong statistical significance. The 95% confidence interval (CI) ranged from 0.814 to 0.870. Heterogeneity was high, with a tau value of 0.071 and an *I*^2^ of 99.57%.

For perpetration, the alpha coefficient was 0.85 with an SE of 0.0221. The Z value was 38.7, and the *p*-value was less than 0.001. The 95% CI ranged from 0.811 to 0.898. The tau value was 0.058, and the *I*^2^ was 98.82%. Similarly, the victimization alpha coefficient was 0.85 with an SE of 0.0180, a Z value of 47.5, and a *p*-value less than 0.001. The 95% CI ranged from 0.820 to 0.890. The tau value was 0.047, and the *I*^2^ was 97.92%.

The omega global coefficient was found to be 0.90 with an SE of 0.0456. The Z value was 19.7, and the *p*-value was less than 0.001. The 95% CI ranged from 0.811 to 0.990, with a tau value of 0.079 and an *I*^2^ of 99.85%. The psychological perpetration alpha coefficient was 0.80 with an SE of 0.0328. The Z value was 24.3, and the *p*-value was less than 0.001. The 95% CI ranged from 0.730 to 0.859, with a tau value of 0.079 and an *I*^2^ of 98.68%.

The physical perpetration alpha coefficient was 0.82 with an SE of 0.0248. The Z value was 33.1, and the *p*-value was less than 0.001. The 95% CI ranged from 0.770 to 0.867, with a tau value of 0.054 and an *I*^2^ of 98.43%. The sexual perpetration alpha coefficient was 0.69 with an SE of 0.0796. The Z value was 8.7, and the *p*-value was less than 0.001. The 95% CI ranged from 0.536 to 0.848, with a tau value of 0.176 and an *I*^2^ of 99.57%.

For psychological victimization, the alpha coefficient was 0.77 with an SE of 0.0295. The Z value was 26, and the *p*-value was less than 0.001. The 95% CI ranged from 0.710 to 0.826, with a tau value of 0.071 and an *I*^2^ of 97.96%. The physical victimization alpha coefficient was 0.80 with an SE of 0.0222. The Z value was 36.2, and the *p*-value was less than 0.001. The 95% CI ranged from 0.760 to 0.847, with a tau value of 0.048 and an *I*^2^ of 97.75%.

The sexual victimization alpha coefficient was 0.64 with an SE of 0.0907. The Z value was 7.03, and the *p*-value was less than 0.001. The 95% CI ranged from 0.459 to 0.815, with a tau value of 0.201 and an *I*^2^ of 99.55%. The Comparative Fit Index (CFI) was 0.95 with an SE of 0.0123. The Z value was 77.2, and the *p*-value was less than 0.001. The 95% CI ranged from 0.929 to 0.978, with a tau value of 0.049 and an *I*^2^ of 99.99%.

The Tucker–Lewis Index (TLI) was 0.96 with an SE of 0.00826. The Z value was 116, and the *p*-value was less than 0.001. The 95% CI ranged from 0.940 to 0.973, with a tau value of 0.025 and an *I*^2^ of 99.85%. The Root Mean Square Error of Approximation (RMSEA) was 0.033 with an SE of 0.0100. The Z value was 2.31, and the *p*-value was less than 0.001. The 95% CI ranged from 0.014 to 0.053, with a tau value of 0.00 and an *I*^2^ of 0%.

The Standardized Root Mean Square Residual (SRMR) was 0.025 with an SE of 0.0238. The Z value was 1.03, and the *p*-value was less than 0.001. The 95% CI ranged from 0.022 to 0.71, with a tau value of 0.00 and an *I*^2^ of 0%. The results indicated high reliability and significant statistical validation for the instruments measuring dating violence perpetration and victimization, as well as excellent model fit indices for structural validity.

**Figure 2:**
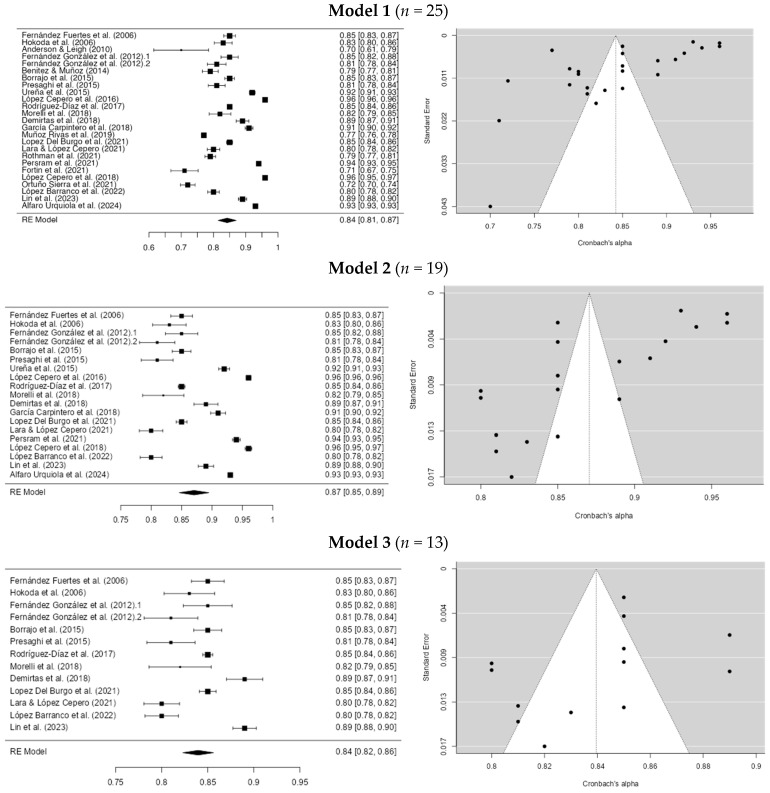

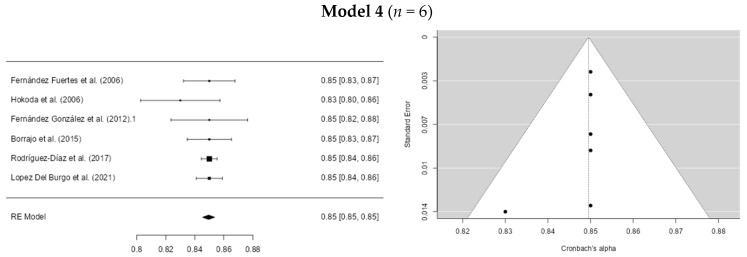
Cronbach’s alpha global sensitivity analysis of dating violence instruments. **Note.** The studies for the model were: Fernández-Fuertes et al. (2006), Hokoda et al. (2006), Anderson & Leigh (2010), Fernández-González et al. (2012), Benítez & Muñoz (2014), Borrajo et al. (2015), Presaghi et al. (2015), Ureña et al. (2015), López-Cepero et al. (2016), Rodríguez-Díaz et al. (2017), Morelli et al. (2018), Demirtas et al. (2018), García-Carpintero et al. (2018), Muñoz-Rivas et al. (2019), López Del Burgo et al. (2021), Lara & López-Cepero (2021), Rothman et al. (2021), Soriano-Ayala et al. (2021), Persram et al. (2021), Fortin et al. (2021), López-Cepero et al. (2018), Ortuño-Sierra et al. (2023), López-Barranco et al. (2022), C. Y. Lin et al. (2023) and Alfaro-Urquiola et al. (2024).

For the dating violence instruments in model 1, the Egger coefficient was −4.466 (*p* > 0.001) and the *I*^2^ was 99.57%. This is the initial model. In the second model, studies with an alpha below 0.80 were eliminated (Anderson & Leigh, 2010; Benítez & Muñoz, 2014; Muñoz-Rivas et al., 2019; Rothman et al., 2021; Ortuño-Sierra et al., 2023; Fortin et al., 2021). Minor differences in the coefficients were found, with Egger = −4.717 (*p* > 0.001) and *I*^2^ = 99.38%. In the third model, studies with an alpha greater than 0.90 were removed (Ureña et al., 2015; Persram et al., 2021; García-Carpintero et al., 2018; López-Cepero et al., 2016, 2018; Alfaro-Urquiola et al., 2024), resulting in coefficients of Egger = −1.607 (*p* = 0.108) and *I*^2^ = 93.68%. In model 4, studies with alpha values lower than 0.83 and higher than 0.85 were eliminated (Fernández-González et al., 2012; Presaghi et al., 2015; Morelli et al., 2018; Demirtas et al., 2018; Lara & López-Cepero, 2021; López-Barranco et al., 2022; C. Y. Lin et al., 2023). We obtained an Egger’s coefficient = −0.744 (*p* = 0.457) and an *I*^2^ coefficient = 0%. Thus, outliers seem to influence the initial model. However, these results should be interpreted with caution. For more information, see Figure 2.

Additionally, it was observed that the most commonly used instruments were CADRI (Conflict in Adolescent Dating Relationship Inventory, Hokoda et al., 2006; Fernández-Fuertes et al., 2006), CADRI-S (Conflict in Adolescent Dating Relationship Inventory short, Fernández-González et al., 2012, study 1), CDAQ (Cyber Dating Abuse Questionnaire, Borrajo et al., 2015), DVQ-R (Dating Violence Questionnaire-Revised, Rodríguez-Díaz et al., 2017), and ADV-YL (Adolescent Dating Violence Questionnaire included in the YourLife Project, Lopez Del Burgo et al., 2021).

**Figure 3:**
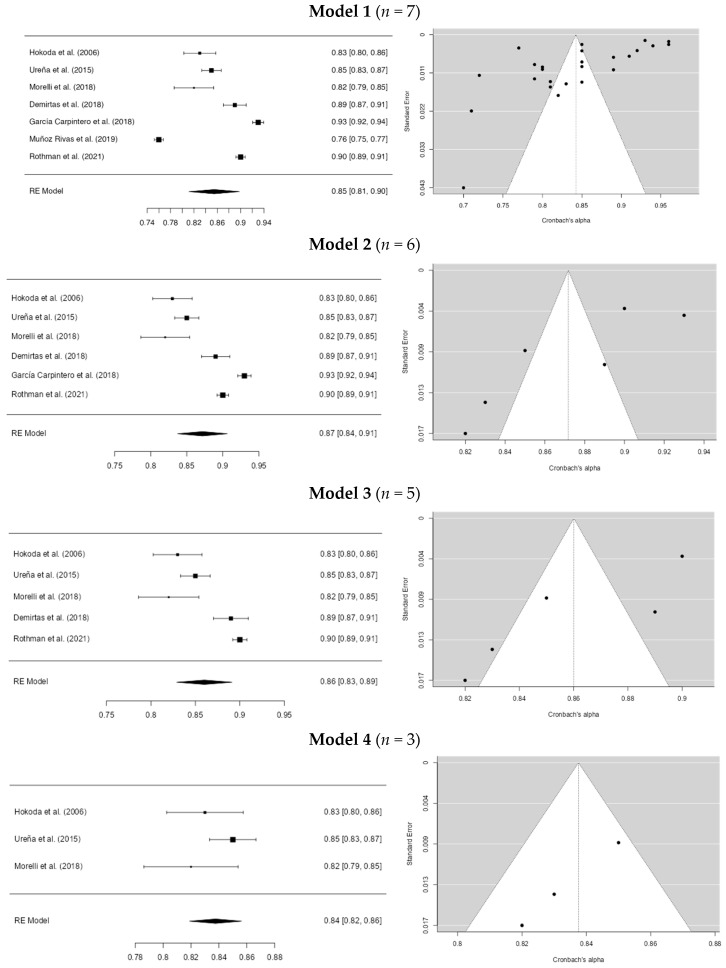
Cronbach’s alpha overall sensitivity analysis of the dating violence instruments in perpetration. **Note.** The studies for the model were: Hokoda et al. (2006), Ureña et al. (2015), Morelli et al. (2018), Demirtas et al. (2018), García-Carpintero et al. (2018) and Rothman et al. (2021).

In the perpetration dating violence instruments for Model 1, Egger’s coefficient was −0.538 (*p* = 0.590), with an *I*^2^ of 98.82%. This is the initial model. In the second model, studies with an alpha lower than 0.80 (Muñoz-Rivas et al., 2019) were removed. Minor differences were found in the coefficients, with Egger = −3.392 (*p* > 0.001) and *I*^2^ = 97.03%. In the third model, studies with an alpha higher than 0.90 (García-Carpintero et al., 2018) were excluded, resulting in coefficients of Egger = −2.733 (*p* = 0.006) and *I*^2^ = 93.40%. In Model 4, studies with alpha values below 0.82 and above 0.85 (Demirtas et al., 2018; Rothman et al., 2021) were excluded. The results showed Egger’s coefficient = −1.793 (*p* = 0.073) and *I*^2^ = 40.52%. Thus, outliers seem to influence the initial model. However, these results should be interpreted with caution. For more information, see Figure 3. Additionally, it was observed that the most commonly used instruments were the CADRI (Conflict in Adolescent Dating Relationship Inventory, Hokoda et al., 2006), the PVD-Q (Psychological Dating Violence Questionnaire, Ureña et al., 2015), and the CDVI (Cyber Dating Violence Inventory, Morelli et al., 2018).

**Figure 4:**
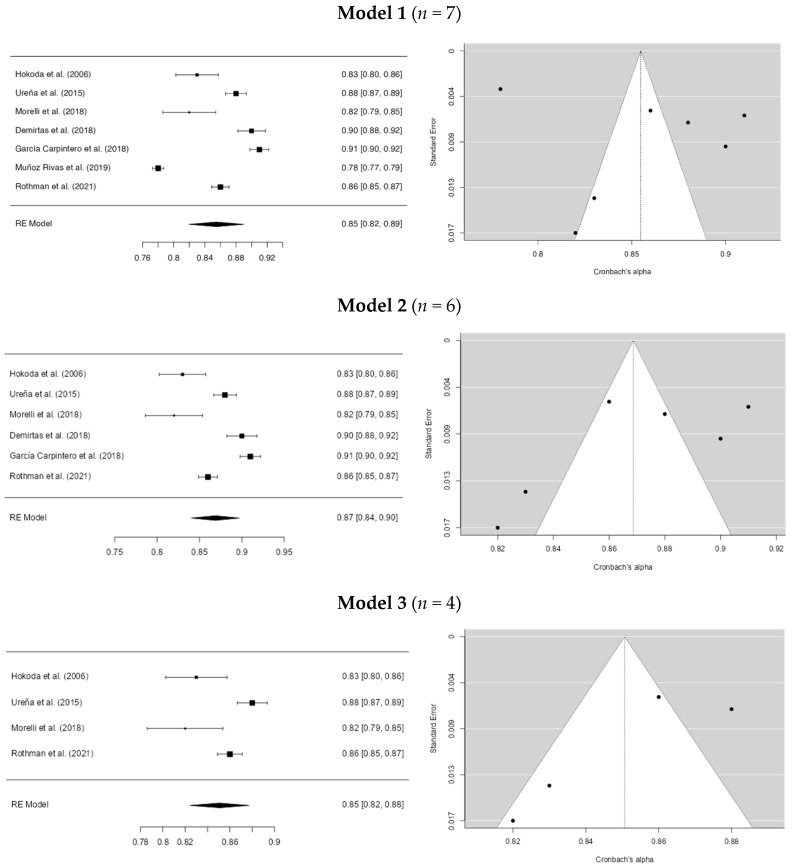

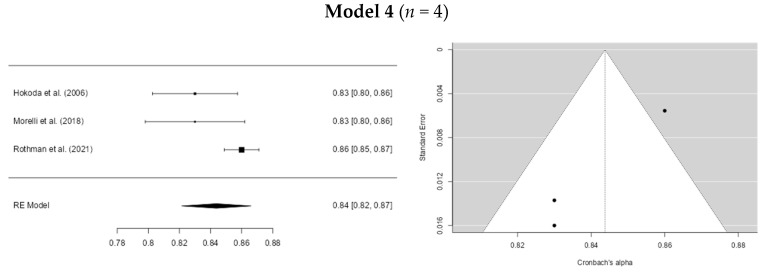
Cronbach’s alpha overall sensitivity analysis of dating violence instruments on victimization. **Note.** The studies for the model were: Hokoda et al. (2006), Ureña et al. (2015), Morelli et al. (2018), Demirtas et al. (2018), García-Carpintero et al. (2018), Muñoz-Rivas et al. (2019) and Rothman et al. (2021).

In the victimization dating violence instruments for Model 1, Egger’s coefficient was −3.334 (*p* = 0.738), with an *I*^2^ of 97.92%. This is the initial model. In the second model, studies with an alpha lower than 0.80 (Muñoz-Rivas et al., 2019) were removed. Minor differences were found in the coefficients, with Egger = −2.305 (*p* = 0.021) and *I*^2^ = 94.62%. In the third model, studies with an alpha higher than 0.90 (Demirtas et al., 2018; García-Carpintero et al., 2018) were excluded, resulting in coefficients of Egger = −2.398 (*p* = 0.016) and *I*^2^ = 88.22%. In Model 4, studies with alpha values below 0.82 and above 0.85 (Ureña et al., 2015) were excluded. The results showed Egger’s coefficient = −2.470 (*p* = 0.014) and *I*^2^ = 64.92%. Thus, outliers seem to influence the initial model. However, these results should be interpreted with caution. For more information, see Figure 4. Additionally, it was observed that the most commonly used instruments were the CADRI (Conflict in Adolescent Dating Relationship Inventory, Hokoda et al., 2006), the CDVI (Cyber Dating Violence Inventory, Morelli et al., 2018), and the MARSHA (Relationship Harassment and Abuse, Rothman et al., 2021).

A total of three eligible studies reported McDonald’s omega (ω), but three measures met the criteria of omega > 0.80 or a sample size with a 10:1 ratio of cases per item (Table 1 and Figure S1). Egger’s regression test indicated significant asymmetry, consistent with potential publication bias (intercept = −2.878, *p* = 0.004). The highest internal reliability estimate was reported by López-Cepero et al. (2018), with an omega of 0.96 (DVQ, 42 items; *n* = 859). However, two other measures yielded acceptable omegas of 0.93 (Alfaro-Urquiola et al., 2024, DVQ-VP, 20 items; *n* = 3776) and 0.81 (Lara & López-Cepero, 2021, DVQ, 42 items; *n* = 846). Additionally, the most commonly used instruments were the Dating Violence Questionnaire (DVQ, López-Cepero et al., 2018; Lara & López-Cepero, 2021) and the Dating Violence Questionnaire for Victimization and Perpetration (DVQ-VP, Alfaro-Urquiola et al., 2024).

In psychological perpetration dating violence instruments, for Model 1, Egger’s coefficient was 0.472 (*p* = 0.637), with an *I*^2^ of 98.68%. This was the initial model (*n* = 6). In the second model, studies with an alpha lower than 0.80 (Fernández-Fuertes et al., 2006; Muñoz-Rivas et al., 2007) were removed. Minor differences were found in the coefficients, with Egger = −0.662 (*p* = 0.508) and *I*^2^ = 85.33%. In the third model, studies with alpha values below 0.82 and above 0.85 (Soriano-Ayala et al., 2021) were removed. The results showed Egger’s coefficient = −0.801 (*p* = 0.423) and *I*^2^ = 0%. Thus, outliers appear to influence the initial model. However, these results should be interpreted with caution. For more information, see Figure S2. Additionally, the most commonly used instruments were the CADRI (Conflict in Adolescent Dating Relationship Inventory, Hokoda et al., 2006), the CDVI (Cyber Dating Violence Inventory, Morelli et al., 2018), and the ADV–YL (Adolescent Dating Violence Questionnaire included in the YourLife Project, Lopez Del Burgo et al., 2021).

In physical perpetration dating violence instruments, for Model 1, Egger’s coefficient was −1.271 (*p* = 0.204), with an *I*^2^ of 98.43%. This was the initial model (*n* = 5). In the second model, studies with an alpha lower than 0.80 (Fernández-Fuertes et al., 2006) were removed. Minor differences were found in the coefficients, with Egger = −0.056 (*p* = 0.956) and *I*^2^ = 96.44%. In the third model, studies with alpha values below 0.81 and above 0.85 (García-Carpintero et al., 2018) were removed. The results showed Egger’s coefficient = −0.001 (*p* = 0.999) and *I*^2^ = 44.51%. Thus, outliers appear to influence the initial model. However, these results should be interpreted with caution. For more information, see Figure S3. Additionally, the most commonly used instruments were the CADRI (Conflict in Adolescent Dating Relationship Inventory, Hokoda et al., 2006), the M-CTS (Modified Version of the Conflicts Tactics Scale, Muñoz-Rivas et al., 2007), and the ADV–YL (Adolescent Dating Violence Questionnaire included in the YourLife Project, Lopez Del Burgo et al., 2021).

In the instruments measuring dating violence in sexual perpetration in Model 1, the Egger coefficient was −5.861 (*p* < 0.001) with an *I*^2^ of 99.57%. This was the initial model (*n* = 5). In the second model, studies with an alpha below 0.70 (Fernández-Fuertes et al., 2006; Hokoda et al., 2006) were removed. Minor differences were found in the coefficients, with Egger = −1.913 (*p* = 0.056) and *I*^2^ = 99.1%. Therefore, outliers seem to influence the initial model. However, these results should be interpreted with caution. For more information, see Figure S4. Additionally, the most commonly used instruments were the MSDV (Multidimensional Scale Dating Violence, García-Carpintero et al., 2018), ADV–YL (Adolescent Dating Violence Questionnaire included in the YourLife Project, Lopez Del Burgo et al., 2021), and TDV-VP (Teen Dating Violence: Victimization and Perpetration Scale, Soriano-Ayala et al., 2021).

For instruments measuring dating violence in psychological victimization in Model 1, the Egger coefficient was 0.257 (*p* = 0.790) with an *I*^2^ of 97.96%. This was the initial model (*n* = 6). In the second model, studies with an alpha below 0.80 (Soriano-Ayala et al., 2021; Fernández-Fuertes et al., 2006; Muñoz-Rivas et al., 2019) were removed. Minor differences were found in the coefficients, with Egger = −1.543 (*p* = 0.123) and *I*^2^ = 40.3%. Therefore, outliers seem to influence the initial model. However, these results should be interpreted with caution. For more information, see Figure S5. Additionally, the most commonly used instruments were the CADRI (Conflict in Adolescent Dating Relationship Inventory, Hokoda et al., 2006), CDVI (Cyber Dating Violence Inventory, Morelli et al., 2018), and ADV–YL (Adolescent Dating Violence Questionnaire included in the YourLife Project, Lopez Del Burgo et al., 2021).

In instruments measuring dating violence in physical victimization in Model 1, the Egger coefficient was −1.931 (*p* = 0.053) with an *I*^2^ of 97.75%. This was the initial model (*n* = 5). In the second model, studies with an alpha below 0.80 (Soriano-Ayala et al., 2021; Hokoda et al., 2006) were removed. Minor differences were found in the coefficients, with Egger = 5.449 (*p* < 0.001) and *I*^2^ = 95.38%. Therefore, outliers seem to influence the initial model. However, these results should be interpreted with caution. For more information, see Figure S6. Additionally, the most commonly used instruments were the MSDV (Multidimensional Scale Dating Violence, García-Carpintero et al., 2018), M-CTS (Modified version of the Conflict Tactics Scale, Muñoz-Rivas et al., 2007), and ADV–YL (Adolescent Dating Violence Questionnaire included in the YourLife Project, Lopez Del Burgo et al., 2021).

For instruments measuring dating violence in sexual victimization in Model 1, the Egger coefficient was −6.920 (*p* < 0.001) with an *I*^2^ of 99.55%. Therefore, outliers seem to influence the initial model. However, these results should be interpreted with caution. For more information, see Figure S7. Additionally, the most commonly used instruments were the MSDV (Multidimensional Scale Dating Violence, García-Carpintero et al., 2018) and ADV–YL (Adolescent Dating Violence Questionnaire included in the YourLife Project, Lopez Del Burgo et al., 2021).

**Figure 5:**
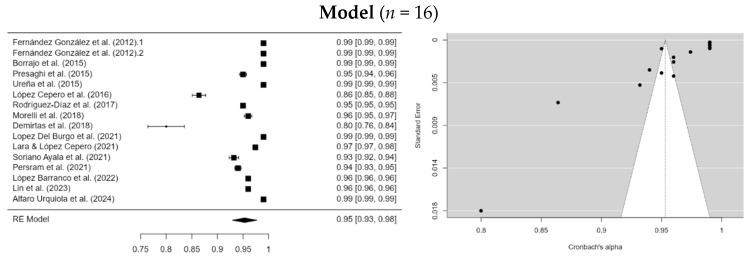
Forest plot of all the studies selected for the meta-analysis of CFI. Note. The research for the model was: Fernández-Fuertes et al. (2012), Borrajo et al. (2015), Presaghi et al. (2015), Ureña et al. (2015), Lopez Del Burgo et al. (2021), López-Cepero et al. (2016), Rodríguez-Díaz et al. (2017), Morelli et al. (2018), Demirtas et al. (2018), Lara & López-Cepero (2021), Soriano-Ayala et al. (2021), Persram et al. (2021), López-Barranco et al. (2022), C. Y. Lin et al. (2023) and Alfaro-Urquiola et al. (2024).

A random-effects meta-analysis was conducted to synthesize values from 16 studies that reported the Comparative Fit Index (CFI) (Figure 5). The results show a cumulative CFI estimate of 0.953 (SE = 0.0122) with a confidence interval (95% CI = 0.929–0.977), indicating a good model fit. However, the statistics (*I*^2^ = 99.98%, Egger = −9.528, *p* < 0.001) and (Q = 3310.959, *p* < 0.001) indicate significant heterogeneity among the studies, attributable to real differences rather than chance.

**Figure 6:**
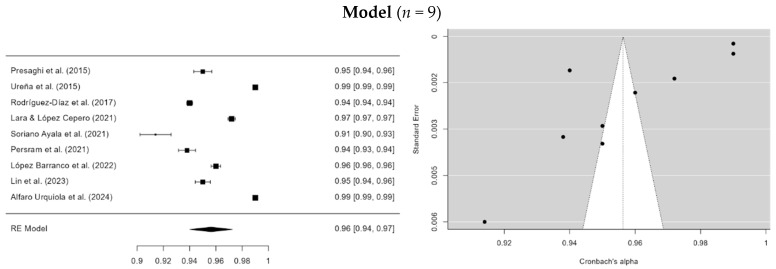
Forest plot of all the studies selected for the meta-analysis of TLI. **Note.** The research for the final model was: Presaghi et al. (2015), Ureña et al. (2015), Rodríguez-Díaz et al. (2017), Lara & López-Cepero (2021), Soriano-Ayala et al. (2021), Persram et al. (2021), López-Barranco et al. (2022), C. Y. Lin et al. (2023) and Alfaro-Urquiola et al. (2024).

A random-effects meta-analysis was conducted to synthesize values from 9 studies that reported the Tucker–Lewis Index (TLI). The results show a cumulative TLI estimate of 0.956 (SE = 0.0826) with a confidence interval (95% CI = 0.940–0.973), indicating a good model fit for the meta-analysis (Figure 6). However, the statistics (*I*^2^ = 99.85%, Egger = −4.195, *p* < 0.001) and (Q = 2948.892, *p* < 0.001) indicated high heterogeneity among the studies, attributable to real differences rather than chance.

Additionally, two random-effects meta-analyses were conducted to evaluate model fit using the RMSEA and SRMR values in instruments measuring dating violence. The first meta-analysis, which included 16 studies, synthesized the values of the Root Mean Square Error of Approximation (RMSEA). The results showed a cumulative RMSEA estimate of 0.0332 (SE = 0.0100) with a 95% confidence interval (CI) ranging from 0.014 to 0.053, indicating that the model fits the observed data well. Furthermore, the statistics *I*^2^ = 0% and Q = 15.000 (*p* < 0.001) indicated moderate heterogeneity among the studies.

The second meta-analysis, which included four studies, synthesized the values of the Standardized Root Mean Square Residual (SRMR). The results showed a cumulative SRMR estimate of 0.0246 (SE = 0.0238) with a 95% CI ranging from −0.022 to 0.071, suggesting a moderately acceptable fit relative to the recommended cutoff point of 0.08. The statistics *I*^2^ *=* 0% and *Q* = 0.736 (*p* < 0.001) also indicated moderate heterogeneity. Similar to the first meta-analysis, Egger’s funnel plot did not reveal publication bias (Figure S8).

## 4. Discussion

The research presented provides a comprehensive overview of various studies on the validation of instruments designed to measure dating violence among adolescents and young adults. These studies, conducted between 2006 and 2024, were published in international psychology and health journals, highlighting the global relevance of this topic. The systematic review selected 26 studies focused on dating violence instruments, addressing aspects such as authors, titles, journals, countries, participant ages, sample sizes, instruments used, number of factors and items, and reliability and structural validity statistics.

Studies conducted in Europe, such as those by Fernández-Fuertes et al. (2006), assessed adolescent dating violence using the CADRI in a sample of 572 adolescents (mean age 16.7 years), reporting an overall alpha of 0.85 and specific alphas for different types of violence ranging from 0.56 (sexual violence) to 0.79 (psychological victimization). Similarly, Muñoz-Rivas et al. (2007) validated a modified version of the Conflict Tactics Scale (M-CTS) with a large sample of 5355 young adults (mean age 19.67 years), obtaining alphas ranging from 0.645 to 0.819 for various types of violence.

Other studies in Spain, such as those by Rodríguez-Díaz et al. (2017) and López-Barranco et al. (2022), also demonstrated high levels of reliability and validity using different instruments. Presaghi et al. (2015) in Italy validated the Italian version of the Dating Violence Questionnaire (DVQ) with a sample of 418 young adults (mean age 22 years), reporting an overall alpha of 0.81 and good model fit indices (CFI = 0.95, TLI = 0.95, RMSEA = 0.033). More recently, Morelli et al. (2018) in Italy validated the Cyber Dating Violence Inventory (CDVI) with 241 young adults (mean age 18.17 years), achieving an overall alpha of 0.82 and strong fit indices (CFI = 0.96, RMSEA = 0.079).

Furthermore, research conducted in Latin America revealed notable efforts to validate dating violence instruments. Hokoda et al. (2006) conducted a study in Mexico using the CADRI with 307 adolescents (mean age 17 years), reporting an overall Cronbach’s alpha of 0.83. López-Cepero et al. (2018) in Peru validated the Digital Intimate Partner Violence Questionnaire (DIPVQ) with 449 young adults (mean age 21.2 years), obtaining an impressive overall alpha and omega of 0.96. In Bolivia, Alfaro-Urquiola et al. (2024) validated the DVQ-VP scale with a large sample of 3776 university students (mean age 20.35 years), reporting an overall alpha of 0.93.

In North America, Anderson and Leigh (2010) in the United States validated the revised Conflict Tactics Scale (CTS2) with 100 deaf female university students (mean age 19 years), achieving an overall alpha of 0.70. Rothman et al. (2021), also in the U.S., validated the MARSHA scale with a nationally representative sample of 1257 young adults (mean age 18 years), reporting alpha values of 0.90 for perpetration and 0.86 for victimization.

In Canada, Fernández-González et al. (2012) developed and validated a short version of the CADRI (CADRI-S) through two studies involving samples of 1277 and 365 adolescents (mean age 15.93 years), with overall alphas of 0.85 and 0.81, respectively. Similarly, Persram et al. (2021) developed and validated the Teen Dating Aggression Measure (TeDAM) with 730 Canadian youth (mean age 15.89 years), achieving an overall alpha of 0.94.

In Asia, C. Y. Lin et al. (2023) in China validated the Dating Violence Bystander Help Intention Questionnaire (DVBHIQ) with a sample of 622 young adults (mean age 20.50 years), reporting an overall alpha of 0.89 and strong structural fit indices (CFI = 0.96, TLI = 0.95, RMSEA = 0.07).

Overall, most studies demonstrated high reliability coefficients (Cronbach’s alpha) and good structural fit indices (CFI, TLI, RMSEA), indicating that the instruments used to assess dating violence are reliable and valid across various cultural contexts and age groups. However, some areas for improvement were identified, particularly in the evaluation of sexual violence, where some studies reported lower alpha coefficients. The diversity of instruments and contexts underscores the need for continuous validation and adaptation of measurement tools to ensure their accuracy and applicability to different populations.

Fernández-Fuertes et al. (2006) and Hokoda et al. (2006) were pioneers in assessing dating violence in adolescent relationships in Spain and Mexico, respectively, using the Conflict in Adolescent Dating Relationships Inventory (CADRI). Both studies found high reliability coefficients, with alphas above 0.80, indicating good internal consistency of the instrument. These studies laid the foundation for subsequent research in the validation of instruments to measure dating violence.

In Spain, Muñoz-Rivas et al. (2007) validated a modified version of the Conflict Tactics Scale (M-CTS) with an extensive sample of 5355 young people, highlighting the robustness of the instrument in measuring violence. This study demonstrated that the M-CTS is an effective tool for assessing different forms of dating violence among youth.

Anderson and Leigh (2010) applied the Revised Conflict Tactics Scales to a sample of deaf college students in the U.S., showing alphas ranging from 0.70 to 0.92, emphasizing the reliability of the instrument in diverse contexts. This study is particularly significant because it addresses a specific, under-studied population, underscoring the importance of adapting and validating instruments for different demographic groups.

In Canada, Fernández-González et al. (2012) developed and validated a shortened version of the CADRI, obtaining alphas of 0.81 and 0.85 in different samples, which strengthens the applicability of this questionnaire to the adolescent population. This abbreviated version makes it easier to administer the questionnaire without compromising its reliability.

Similarly, Benítez and Muñoz (2014) analyzed the CADRI in Spanish college students, reporting high reliability with an alpha of 0.79. This study emphasizes the importance of continuous validation of instruments in different educational and demographic contexts.

Subsequent studies, such as those by Borrajo et al. (2015) and Presaghi et al. (2015), continued exploring dating violence in young couples through new instruments like the Cyber Dating Abuse Questionnaire (CDAQ) and the Dating Violence Questionnaire (DVQ), respectively. Both studies reported high reliability levels and good fit indices, highlighting the validity of these new instruments in assessing contemporary forms of violence, including digital abuse.

Ureña et al. (2015) and López-Cepero et al. (2016) contributed to the validation of questionnaires in different cultural contexts, including the U.S. and Spain, finding Cronbach’s alpha coefficients ranging from 0.85 to 0.96, demonstrating the internal consistency of these instruments. These studies highlight the importance of adapting and validating measurement tools in diverse cultural contexts to ensure their applicability and accuracy.

Recently, studies such as those by Lopez Del Burgo et al. (2021) in Chile, Ecuador, and Spain, and Soriano-Ayala et al. (2021) in Spain, have developed and validated new scales, such as the ADV-YL and the TDV-VP, with high reliability and fit indices, indicating their effectiveness in measuring dating violence in adolescent relationships. These works emphasize the need for specific tools to assess violence dynamics in different cultural and social contexts. In Latin America, Alfaro-Urquiola et al. (2024) validated the DVQ-VP scale in Bolivia, showing an alpha of 0.93 and good fit indices, highlighting the importance of having valid and reliable instruments to assess dating violence in different cultural contexts. This study is an example of the growing attention to dating violence in Latin America and the importance of having suitable assessment tools.

Additionally, studies like those by C. Y. Lin et al. (2023) in China and Fortin et al. (2021) in Canada demonstrate the global expansion of this research, with instruments such as the DVBHIQ and RSI-A addressing violent behaviors and relational skills in adolescents. These studies not only validate the instruments in new contexts but also explore additional dimensions of youth relationships. Rothman et al. (2021) in the U.S. and López-Cepero et al. (2018) in Peru have made significant contributions to the validation of instruments such as MARSHA and DIPVQ, respectively. These studies emphasize the importance of measuring both harassment and digital violence in adolescent relationships, areas that are becoming increasingly relevant in the context of online interactions. Persram et al. (2021) and Morelli et al. (2018) have also provided valuable insights through the validation of new instruments like TeDAM and CDVI in Canada and Italy, respectively. These studies reinforce the importance of having precise tools to assess aggression and victimization in youth relationships.

Finally, Ortuño-Sierra et al. (2023) and López-Barranco et al. (2022), by validating the EAV and CADRI in Spain, have advanced the understanding of attitudes toward violence and the ongoing validation of widely used instruments, ensuring their relevance and reliability in current youth populations. These studies, with their various approaches and geographic contexts, demonstrate a concerted effort to understand and measure dating violence in adolescent and young adult relationships, using validated and reliable tools. The high internal consistency and good fit indices reported highlight the usefulness of these instruments in research and intervention in this field. However, when reviewing these studies and within the framework of a meta-analysis, it is essential to explicitly discuss the strengths and weaknesses of the measures used. While the instruments have shown strong reliability, cultural and contextual differences may influence how violent behaviors are expressed and perceived. Moreover, it is crucial to examine whether the fit indices always reflect an adequate measurement of the variables across different populations and contexts. For example, while the CADRI is robust, it could benefit from further adaptation to more accurately capture the specifics of dating violence in particular cultural contexts. This more detailed analysis of the strengths and limitations of the tools used would enhance the contribution of these studies, providing a more nuanced understanding of their applicability and areas for improvement in future research.

## 5. Limitations

This study, despite its significant findings, has several limitations that should be considered. First, the high heterogeneity observed in the results suggests that the differences between the included studies may have influenced the reported reliability and validity coefficients. This variability could be due to differences in the samples, cultural contexts, or methodologies used in each individual study.

Second, although the instruments demonstrated high reliability overall, the lower coefficients for measuring sexual violence indicate a potential need to improve accuracy and consistency in this specific area. This suggests that current instruments may not fully capture the complexity of sexual violence in dating, which limits the generalizability of these results.

Third, the use of a random-effects model, while suitable for handling heterogeneity, also means that the results should be interpreted with caution. This model assumes that variations between studies are random, which may not always be the case, and this could affect the robustness of the conclusions. Fourth, the reliance on published studies may introduce publication bias, where studies with non-significant or unfavorable results are less likely to be published. This could lead to an overestimation of the reliability and validity of the evaluated instruments. Finally, most of the included studies did not provide detailed data on the demographic characteristics of the samples, which limits the ability to assess how factors such as age, gender, and ethnicity might influence the reliability and validity of the instruments. These limitations highlight the need for future research to address these points, using more diverse samples and robust methodologies to ensure the generalization and accuracy of dating violence measurement instruments.

## 6. Conclusions

In conclusion, the evaluated instruments demonstrated high reliability and structural validity in measuring perpetration and victimization of dating violence. The high global alpha and omega coefficients, along with excellent model fit indices, underscore the strength and consistency of these tools across various applications. However, areas for improvement were identified, particularly in the measurement of sexual violence, where the coefficients were lower compared to other types of violence.

Heterogeneity statistics indicated significant variability between studies, suggesting the need to consider diverse contexts and samples in future research. Despite this variability, the overall consistency of the instruments was notable, supporting their continued use both in research and clinical practice. However, the manuscript acknowledges the significant heterogeneity in psychometric properties across all studies, but the discussion of potential explanations for these differences is limited. A deeper exploration of factors such as cultural differences, sample characteristics, and methodological variations should be considered to better understand this variability. For example, cultural factors could influence how certain items are interpreted or how respondents perceive the instruments. Similarly, sample characteristics such as age, gender, and socio-economic status could impact the reliability and validity of the instruments. Methodological differences, including variations in data collection procedures or the statistical methods used, may also contribute to discrepancies in the psychometric properties observed. Addressing these factors in future research would help clarify the reasons for the observed heterogeneity and improve the generalizability of the findings.

These findings reinforce confidence in the current instruments to effectively measure dating violence, while highlighting the importance of continuing to refine and adapt these tools to ensure their accuracy and relevance in all contexts. The validation of these instruments makes a significant contribution to the field, providing reliable tools for identifying and analyzing violence in young romantic relationships. Additionally, the suggested recent studies (Commodari et al., 2024; Görgülü & Özer, 2024; Liu et al., 2023) have been cited to strengthen the discussion on adolescent social behaviors and online interactions, providing a broader context for evaluating dating violence and online victimization. These studies shed light on the influence of parental mediation, social skills, and internet use on adolescents, contributing to a deeper understanding of the complex dynamics surrounding dating violence in both offline and online environments.

Future research should focus on further expanding the psychometric evaluation of instruments for measuring both perpetration and victimization of dating violence, particularly by assessing their validity and reliability across diverse cultural, social, and economic contexts. This would allow for a more nuanced understanding of how these factors influence the accuracy of these tools. Furthermore, longitudinal studies could be valuable to explore the evolution of dating violence in adolescents, specifically examining the relationship between early experiences of violence and subsequent risks of perpetration or victimization in later relationships. Such research would provide a stronger basis for identifying risk and protective factors, thereby enhancing the development of targeted prevention interventions.

Incorporating new technologies, such as virtual reality and big data analytics, into future studies could offer innovative ways to capture the complexities of dating violence in both offline and online settings. These technologies could provide a more comprehensive understanding of adolescent relationships, particularly in the digital age, where online interactions are increasingly integral to romantic relationships. Additionally, investigating the role of parental guidance, social skills, and digital literacy in mitigating online victimization could further illuminate the mechanisms that drive adolescent engagement in violent behaviors in both real and virtual environments. These expanded perspectives will not only enhance the reliability of dating violence instruments but also contribute to the development of more effective, contextually relevant interventions.

## Figures and Tables

**Table 1:** Characteristics of the articles selected for the study on dating violence (*n* = 26).

Study	Authors	Journal	Country	Age	Sample	Instrument	*n* Factors	*n* Items	Reliability y Structural Validity
1	Fernández-Fuertes et al. (2006)	International Journal of Clinical and Health Psychology	Spain	M: 16.7 Range: 15–19	572	CADRI	2	25	α Global: 0.85 α Psychological Perpetration: 0.78 α Physical Perpetration: 0.73 α Sexual Perpetration: 0.56 α Psychological Victimization: 0.79 α Physical Victimization: 0.76 α Sexual Victimization: 0.56
2	Hokoda et al. (2006)	Violence and Victims	México	M: 17 Range: 15–18	307	CADRI	2	34	α Global: 0.83 α Perpetration: 0.83 α Psychological Perpetration: 0.82 α Physical Perpetration: 0.82 α Sexual Perpetration: 0.46 Victimization: 0.83 α Psychological Victimization: 0.80 α Physical Victimization: 0.74 α Sexual Victimization: 0.41
3	Muñoz-Rivas et al. (2007)	Psicothema	Spain	M: 19.67 Range: 16–26	5355	M-CTS	2	18	α Psychological Perpetration: 0.645 α Physical Perpetration: 0.819 α Psychological Victimization: 0.645 α Physical Victimization: 0.813
4	Anderson and Leigh (2010)	Journal of Family Violence	United States	M: 19 Range: 18–25	100	CTS 2	2	35	α Global: 0.70 α Psychological Perpetration: 0.71 α Physical Perpetration: 0.75 α Sexual Perpetration: 0.42 α Psychological Victimization: 0.80 α Physical Victimization: 0.92 α Sexual Victimization: 0.65
5	Fernández-González et al. (2012)	Advances in Mental Health	Canada	Study 1 M: 15.93 Range: 13–19 Study 2 M: 15.93 Range: 13–18	Study 1: 1277 Study 2: 365	CADRI-S	2	10	Study 1: α Global: 0.85 Study 2: α Global: 0.81 Study 1 and 2: CFI: 0.99 RMSEA: 0.00
6	Benítez and Muñoz (2014)	Universitas Psychologica	Spain	M: 18.76 Range: 17–21	571	CADRI	2	59	α Global: 0.79 α Psychological Perpetration: 0.80 α Physical Perpetration: 0.81 α Psychological Victimization: 0.81 α Physical Victimization: 0.75 CFI: 0.936 RMSEA: 0.026
7	Borrajo et al. (2015)	Computers in Human Behavior	Spain	M: 22.72 Range: 18–30	788	CDAQ	2	20	α Global: 0.85 CFI: 0.99 RMSEA: 0.076
8	Presaghi et al. (2015)	PLoS One	Italy	M: 22 Range: 16–26	418	DVQ	8	42	α Global: 0.81 CFI: 0.95 TLI: 0.95 RMSEA: 0.033
9	Ureña et al. (2015)	International Journal of Clinical and Health Psychology	Spain	M: 22 Range: 19–25	670	PVD-Q	2	15	α Global: 0.92 α Perpetration: 0.85 α Victimization: 0.88 CFI: 0.99 TLI: 0.99 RMSEA: 0.06
10	López-Cepero et al. (2016)	Violence and Victims	United States	M: 19 Range: 18–26	859	DVQ	8	42	α Global: 0.96 CFI: 0.864 RMSEA: 0.064
11	Rodríguez-Díaz et al. (2017)	International Journal of Clinical and Health Psychology	Spain	M: 18.5 Range: 15–26	6138	DVQ-R	5	20	α Global: 0.85 CFI: 0.95 TLI: 0.94 RMSEA: 0.018
12	Morelli et al. (2018)	European Journal of Developmental Psychology	Italy	M: 18.17 Range: 13–22	241	CDVI	2	11	α Global: 0.82 α Perpetration: 0.82 α Psychological Perpetration: 0.82 α Psychological Victimization: 0.82 CFI: 0.96 RMSEA: 0.079
13	Demirtas et al. (2018)	Violence and Victims	Turkey	M: 21.34 Range: 18–28	254	MMEA	2	28	α Global: 0.89 α Perpetration: 0.89 α Victimization: 0.90 CFI: 0.80 RMSEA: 0.07 SRMS: 0.07
14	García-Carpintero et al. (2018)	Sanitary Gazette	Spain	Range: 18–24	447	MSDV	2	32	α Global: 0.91 α Perpetration: 0.93 α Physical Perpetration: 0.888 α Sexual Perpetration: 0.888 α Victimization: 0.91 α Physical Victimization: 0.865 α Sexual Victimization: 0.865
15	Muñoz-Rivas et al. (2019)	Anales de Psicología	Spain	M: 19.11 Range: 14–26	8105	DJTS	2	11	α Global: 0.77 α Perpetration: 0.76 α Victimization: 0.78 CFI: 0.977 RMSEA: 0.03
16	Lopez Del Burgo et al. (2021)	International Journal of Environmental Research and Public Health	Chile, Ecuador and Spain	M: 15.9 Range: 13–18	2254	ADV–YL	2	18	α Global: 0.85 α Psychological Perpetration: 0.83 α Physical Perpetration: 0.83 α Sexual Perpetration: 0.83 α Psychological Victimization: 0.83 α Physical Victimization: 0.83 α Sexual Victimization: 0.83 CFI: 0.990 RMSEA: 0.031 SRMS: 0.01
17	Lara and López-Cepero (2021)	Journal of interpersonal violence	Chile	M: 17.87 Range: 14–24	846	DVQ	8	42	α Global: 0.80 ω Global: 0.81 CFI: 0.974 TLI: 0.972 RMSEA: 0.02
18	Rothman et al. (2021)	Journal of Interpersonal Violence	United States	M: 18 Range: 11–21	1257	MARSHA	2	39	α Global: 0.79 α Perpetration: 0.90 α Victimization: 0.86
19	Soriano-Ayala et al. (2021)	International Journal of Environmental Research and Public Health	Spain	M: 18 Range: 13–21	422	TDV-VP	2	25	α Psychological Perpetration: 0.874 α Sexual Perpetration: 0.707 α Psychological Victimization: 0.722 α Sexual Victimization: 0.503 CFI: 0.932 TLI: 0.914 RMSEA: 0.07
20	Persram et al. (2021)	Frontiers in psychology	Canada	M: 15.89 Range: 11–18	730	TeDAM	3	44	α Global: 0.94 CFI: 0.94 TLI: 0.938 RMSEA: 0.057
21	Fortin et al. (2021)	Journal of adolescence	Canada	M: 16.11 Range: 14–19	384	RSI-A	3	16	α Global: 0.71 CFI: 0.90 RMSEA: 0.06 SRMR: 0.06
22	López-Cepero et al. (2018)	Journal of interpersonal violence	Perú	M: 21.2	449	DIPVQ	2	12	α Global: 0.96 ω Global: 0.96 SRMR: 0.03
23	Ortuño-Sierra et al. (2023)	International journal of environmental research and public health	Spain	M: 16.12 Range: 13–21	1305	EAV	2	10	α Global: 0.72 ω Global: 0.76 CFI: 0.95 TLI: 0.95 RMSEA: 0.06
24	López-Barranco et al. (2022)	International journal of environmental research and public health	Spain	M: 21.7 Range: 19–25	976	CADRI	6	34	α Global: 0.80 CFI: 0.96 TLI: 0.96 RMSEA: 0.04
25	C. Y. Lin et al. (2023)	Heliyon	China	M: 20.50 Range: 18–23	622	DVBHIQ	4	17	α Global: 0.89 CFI: 0.96 TLI: 0.95 RMSEA: 0.07 SRMR: 0.05
26	Alfaro-Urquiola et al. (2024)	International Journal of Psychological Research	Bolivia	M: 20.35 Range: 17–25	3776	DVQ-VP	5	20	α Global: 0.93 ω Global: 0.93 CFI: 0.99 TLI: 0.99 RMSEA: 0.02

**Note:** M = Median age, α = Cronbach’s alpha, ω = McDonald’s omega, CFI = Comparative Fit Index, TLI = Tucker–Lewis Index, RMSEA = Root Mean Square Error of Approximation, SRMR = Standardized Root Mean Square Residual.

**Table 2:** Descriptives of the dating violence instruments used.

Instrument	Abbreviation	*n* (%)
Adolescent dating violence questionnaire included in the YourLife project	ADV–YL	1 (3.7%)
Conflict in Adolescent Dating Relationship Inventory	CADRI	4 (15%)
Conflict in Adolescent Dating Relationship inventory’ short	CADRI-S	2 (7.4%)
Cyber Dating Abuse Questionnaire	CDAQ	1 (3.7%)
Cyber dating violence inventory	CDVI	1 (3.7%)
Revised Conflict Tactics Scales	CTS 2	1 (3.7%)
Digital Intimate Partner Violence Questionnaire	DIPVQ	1 (3.7%)
Dominating and Jealous Tactics Scale	DJTS	1 (3.7%)
Dating Violence Bystander Help Intention Questionnaire	DVBHIQ	1 (3.7%)
Dating Violence Questionnaire	DVQ	3 (11%)
Dating Violence Questionnaire-Revised	DVQ-R	1 (3.7%)
Dating Violence Questionnaire for Victimization and Perpetration	DVQ-VP	1 (3.7%)
Attitudes Scale towards Intimate Violence	EAV	1 (3.7%)
Modified version Conflicts Tactics Scale	M-CTS	1 (3.7%)
Relationship Harassment and Abuse	MARSHA	1 (3.7%)
Multidimensional measure of emotional abuse	MMEA	1 (3.7%)
Multidimensional Scale Dating Violence	MSDV	1 (3.7%)
Psychological Dating Violence Questionnaire	PVD-Q	1 (3.7%)
Relational Skills Inventory for Adolescents	RSI-A	1 (3.7%)
Teen Dating Violence: Victimization and Perpetration scale	TDV-VP	1 (3.7%)
Teen Dating Aggression Measure Among Canadian Youth	TeDAM	1 (3.7%)

**Note.** For better visualization, see the following link in additional Figure Additional 1: https://goo.su/9YHLvW.

**Table 3:** Summary measures of sample, items, reliability, and fit measures.

	*n*	No Report	Medium	SD	Minimum	Maximum
Average age	26	1	18.74	2.19	15.89	22.72
Min Age	26	1	15.34	2.46	11	19
Max Age	26	1	22.84	3.47	18	30
Sample	27	0	1425.48	2020.92	100	8.105
*n* items	27	0	25.51	13.22	10	59
α Global	25	2	0.84	0.07	0.70	0.96
α Perpetration	7	20	0.85	0.05	0.76	0.93
α Victimization	7	20	0.85	0.04	0.78	0.91
α Psychological Perpetration	8	19	0.78	0.07	0.64	0.87
α Physical Perpetration	7	20	0.80	0.05	0.73	0.88
α Sexual Perpetration	6	21	0.64	0.19	0.42	0.88
α Psychological Victimization	8	19	0.77	0.06	0.64	0.83
α Physical Victimization	7	20	0.81	0.06	0.74	0.92
α Sexual Victimization	6	21	0.63	0.18	0.41	0.86
ω Global	4	23	0.86	0.09	0.76	0.96
CFI	20	7	0.95	0.04	0.80	1.00
TLI	10	17	0.95	0.02	0.91	0.99
RMSEA	20	7	0.04	0.02	0.00	0.07
SRMR	5	22	0.04	0.02	0.01	0.07

**Note.** α = Cronbach’s alpha, ω = McDonald’s omega, CFI = Comparative Fit Index, TLI = Tucker–Lewis Index, RMSEA = Root Mean Square Error of Approximation, SRMR = Standardized Root Mean Square Residual.

**Table 4:** Random effects model and heterogeneity statistics informing the reliability and structural validity of dating violence perpetration and victimization instruments.

	E	SE	Z	*p*	CI LB	CI UB	Tau	Tau^2^	*I* ^2^	*H* ^2^	*df*	*Q*	*p*
α Global	0.82	0.0143	58.8	<0.001	0.814	0.870	0.071	0.005 (SE = 0.0015)	99.57%	233.360	24	4.448.972	<0.001
α Perpetration	0.85	0.0221	38.7	<0.001	0.811	0.898	0.058	0.0033 (SE = 0.002)	98.82%	84.432	6	968.300	<0.001
α Victimization	0.85	0.0180	47.5	<0.001	0.820	0.890	0.047	0.0022 (SE = 0.0013)	97.92%	48.019	6	494.995	<0.001
ω Global	0.90	0.0456	19.7	<0.001	0.811	0.990	0.079	0.0062 (SE = 0.0062)	99.85%	646.499	2	267.473	<0.001
α Psychological Perpetration	0.80	0.0328	24.3	<0.001	0.730	0.859	0.079	0.0063 (SE = 0.0041)	98.68%	577.461	5	577.461	<0.001
α Physical Perpetration	0.82	0.0248	33.1	<0.001	0.770	0.867	0.054	0.003 (SE = 0.0022)	98.43%	63.492	4	104.594	<0.001
α Sexual Perpetration	0.69	0.0796	8.7	<0.001	0.536	0.848	0.176	0.0031 (SE = 0.0224)	99.57%	232.336	4	265.517	<0.001
α Psychological Victimization	0.77	0.0295	26	<0.001	0.710	0.826	0.071	0.005 (SE = 0.0033)	97.96%	48.967	5	466.074	<0.001
α Physical Victimization	0.80	0.0222	36.2	<0.001	0.760	0.847	0.048	0.00 (SE = 0.0017)	97.75%	44.434	4	62.251	<0.001
α Sexual Victimization	0.64	0.0907	7.03	<0.001	0.459	0.815	0.201	0.0402 (SE = 0.0291)	99.55%	223.266	4	276.462	<0.001
CFI	0.95	0.0122	78	<0.001	0.929	0.977	0.049	0.0024 (SE = 0.0040)	99.98%	4649.732	15	3319.959	<0.001
TLI	0.96	0.00826	116	<0.001	0.940	0.973	0.025	0.0064 (SE = 0.0034)	99.85%	648.489	8	2948.892	<0.001
RMSEA	0.033	0.0100	2.31	<0.001	0.014	0.053	0.00	0.00 (SE = 0.00)	0%	1.000	15	4.409	*0.996*
SRMR	0.025	0.0238	1.03	<0.001	0.022	0.71	0.00	0.00 (SE = 0.0021)	0%	1.000	3	0.736	*0.865*

**Note:** E = Estimate, SE = Standard Error, Z = Z-score, *p* = *p*-value, CI LB = Confidence Interval Lower Bound, CI UB = Confidence Interval Upper Bound, Tau = Tau coefficient, Tau^2^ = Tau-squared (variance of true effect sizes), *I*^2^ = I-squared (percentage of total variation due to heterogeneity), *H*^2^ = H-squared (relative excess in Q over its degrees of freedom), *df* = Degrees of Freedom, *Q* = Cochran’s Q statistic for heterogeneity.

## Data Availability

The data supporting this research are publicly available and can be obtained by emailing the first author of this article.

## Supplementary Materials

The following supporting information can be downloaded at: https://www.mdpi.com/article/10.3390/ejihpe15040044/s1, Supplementary Table (Table S1. Search Phrases Used for the Systematic Review) and Supplementary Figures of the Meta-Analysis (Figures S1–S8).
