# Supplementary material for: Psychometric Properties of Instruments for Perpetration and Victimization of Dating Violence in Young People: Systematic Review and Meta-Analysis
Source: Eur J Investig Health Psychol Educ. 2025 Mar 24;15(4):44. doi: 10.3390/ejihpe15040044 (PMC12026061; doi:10.3390/ejihpe15040044)
Supplement: Supplementary file 1 [file ejihpe-15-00044-s001.zip › ejihpe-3509813-supplementary.pdf]

Supplementary

Table S1.

Search phrases used for systematic review.

Base	*Términos de la búsqueda*	*n*
**PubMed**	(("Partner Violence Intimate" OR "Violence Intimate Partner" OR "Intimate Partner Abuse" OR "Abuse Intimate Partner" OR "Partner Abuse Intimate" OR "Dating Violence" OR "Violence Dating" OR "gender-based violence" OR "gender-based violence" OR "gender-based violence" OR "gender" OR "based" OR "violence" OR "gender based violence" OR "against" AND "women" OR "violence against women") AND (Adolescents OR Adolescence OR Teens OR Teen OR Teenagers OR Teenager OR Youth OR Youths OR "Adolescents Female" OR "Adolescent Female" OR "Female Adolescent" OR "Female Adolescents" OR "Adolescents Male" OR "Adolescent Male" OR "Male Adolescent" OR "Male Adolescents") AND (Adaptation OR Validation OR Invariance OR psychometric OR "validation studies as topic" OR validation study" OR "validation studies as topic" OR "validation scale" OR instrument))	1746
**Web Of Science**	(Partner Violence Intimate OR Violence Intimate Partner OR Intimate Partner Abuse OR Abuse Intimate Partner OR Partner Abuse Intimate OR Dating Violence OR Violence Dating OR gender-based violence OR gender-based violence OR gender-based violence OR gender OR based OR violence OR gender based violence OR against AND women OR violence against women) AND (Adolescents OR Adolescence OR Teens OR Teen OR Teenagers OR Teenager OR Youth OR Youths OR Adolescents Female OR Adolescent Female OR Female Adolescent OR Female Adolescents OR Adolescents Male OR Adolescent Male OR Male Adolescent OR Male Adolescents) AND (Adaptation OR Validation OR Invariance OR psychometric OR validation studies OR validation study OR validation studies as topic OR validation scale OR instrument)	2898
**PsyINFO**	((Partner Violence Intimate OR Violence Intimate Partner OR Intimate Partner Abuse OR Abuse Intimate Partner OR Partner Abuse Intimate OR Dating Violence OR Violence Dating OR gender-based violence OR gender-based violence OR gender-based violence OR gender OR based OR violence OR gender based violence OR against AND women OR violence against women) AND (Adolescents OR Adolescence OR Teens OR Teen OR Teenagers OR Teenager OR Youth OR Youths OR Adolescents Female OR Adolescent Female OR Female Adolescent OR Female Adolescents OR Adolescents Male OR Adolescent Male OR Male Adolescent OR Male Adolescents) AND (Adaptation OR Validation OR Invariance OR psychometric OR validation studies as topic OR validation study OR validation studies as topic OR validation scale OR instrument))	3008
**Scopus**	TITLE-ABS-KEY ((partner OR violence OR intimate OR abuse AND intimate OR partner AND abuse AND intimate OR dating AND violence OR violence AND dating OR violence OR gender-based AND violence OR gender OR based OR violence OR	2466

	gender) AND (adolescents OR adolescence OR teens OR teen OR teenagers OR teenager OR youth OR youths OR adolescents AND female OR adolescent AND female OR female AND adolescent OR female OR male AND adolescent OR male AND adolescents) OR (adaptation OR validation OR invariance OR psychometric OR validation AND studies OR validation AND study OR validation AND studies AND as AND topic OR validation AND scale OR instrument))
**Total articles**	10118

Note: *Date of the search: June 27th, 2024. In the Pubmed and PsycINFO databases, the search was conducted in "all fields," identifying specific terms simultaneously in titles, abstracts, and full texts. In Scopus, the search was conducted in the fields of title, abstract, and author-specified keywords. In Web of Science, the search was performed in the "topic" field to identify terms appearing in titles, abstracts, and keyword indexing.*

The search string was customized according to each database to comprehensively reach the literature, given the variability in database structures and indexing methods. The following filters were applied to the search results: document type: "article" in PsycINFO and Scopus, and "academic publication" in Web of Science. Boolean operators "AND" and "OR" were used in all four databases.

Figure S1.

Forest plot of the reliability coefficients in the articles that globally reported McDonald's omega (n = 3).

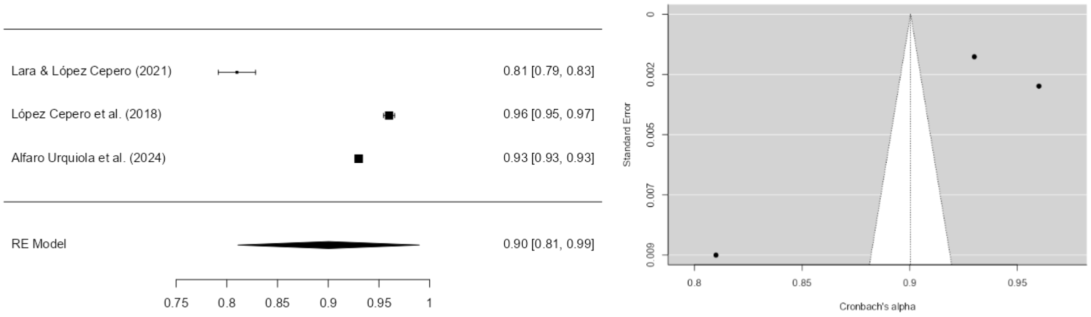

A total of three eligible studies reported McDonald's omega (ω), but three measures were found that met the criteria of an omega > 0.80 or a sample size of 10:1 cases-to-items ratio (Table 1 and Figure S1). The Egger regression test indicated significant asymmetry, which is consistent with potential publication bias (intercept = −2.878, p = 0.004). The highest internal reliability estimate was reported by López Cepero et al. (2018), with an omega of .96 (DVQ, 42 items; n = 859). However, the two other measures yielded acceptable omegas of .93 (Alfaro Urquiota et al., 2024, DVQ-VP, 20 items; n = 3776) and .81 (Lara and López Cepero et al., 2021, DVQ, 42 items; n = 846). Additionally, it was observed that the most commonly used instruments were the Dating Violence Questionnaire (DVQ, López Cepero et al., 2018; Lara and López Cepero et al., 2021) and the Dating Violence Questionnaire for Victimization and Perpetration (DVQ-VP, Alfaro Urquiota et al., 2024).

Figure S2.

Cronbach's alpha overall sensitivity analysis of the dating violence instruments in psychological perpetration.

Model 1 (*n* = 6)

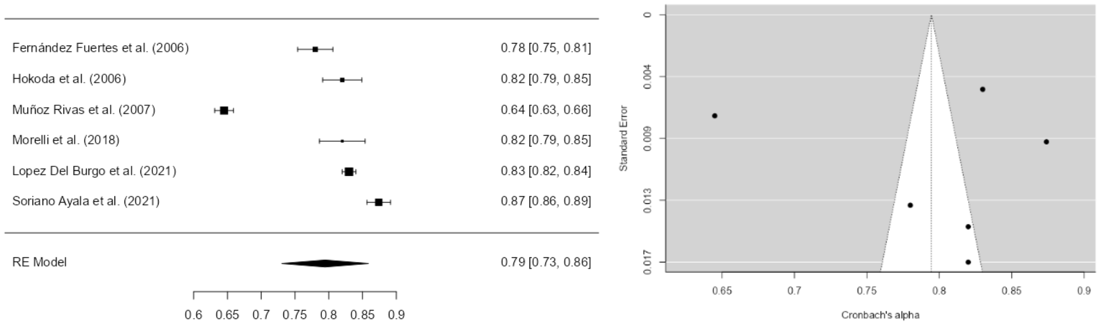

Model 2 (*n* = 4)

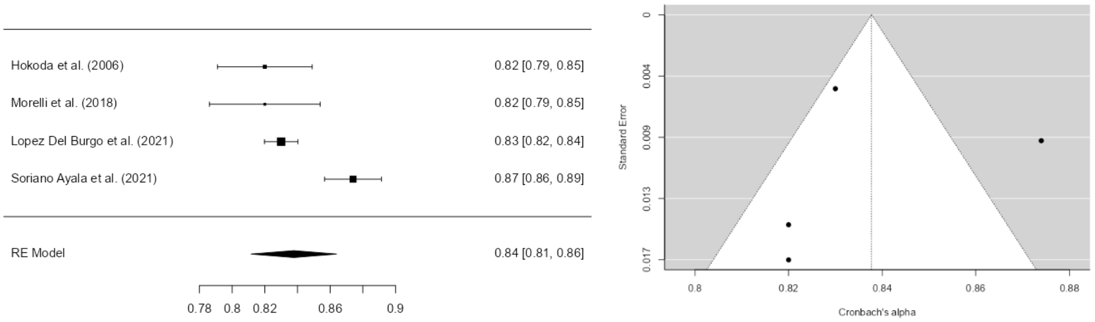

Model 3 (*n* = 3)

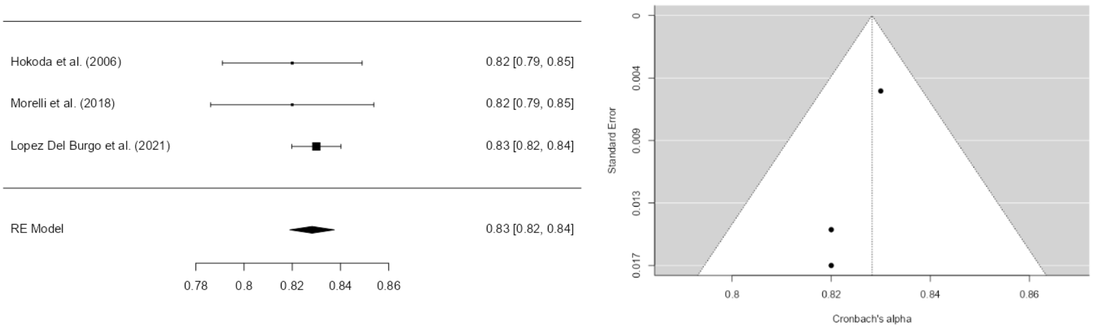

In the dating violence instruments for psychological perpetration in Model 1, the Egger coefficient was 0.472 (p = 0.637) and the I^2 was 98.68%. This is the initial model (n = 6). In the second model, studies with an alpha lower than .80 (FernándezFuertes et al., 2006; MuñozRivas et al., 2007) were excluded. Minor differences were found in the coefficients, with Egger = −0.662 (p = 0.508) and I^2 = 85.33%. In the third model, studies with alpha values lower than 0.82 and higher than 0.85 (Soriano Ayala et al., 2021) were excluded. The Egger coefficient was −0.801 (p = 0.423) and the I2 coefficient was 0%. Thus, outliers appear to influence the initial model. However, these results should be interpreted with caution. For further details, refer to Figure S2. Additionally, the most commonly used instruments were CADRI (Conflict in Adolescent Dating Relationship Inventory, Hokoda et al., 2006), CDVI (Cyber Dating Violence Inventory, Morelli et al., 2018), and ADV–YL (Adolescent Dating Violence Questionnaire included in the YourLife Project, Lopez Del Burgo et al., 2021).

Figure S3.

Cronbach's alpha overall sensitivity analysis of the dating violence instruments in physical perpetration.

Model 1 (*n* = 5)

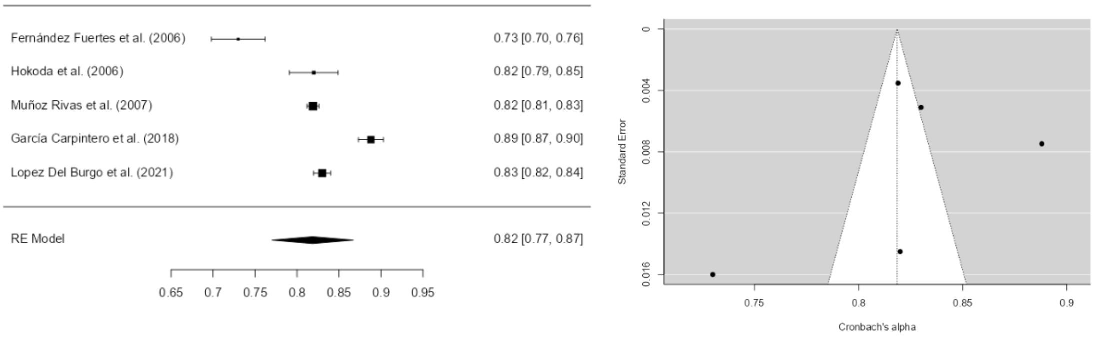

Model 2 (*n* = 4)

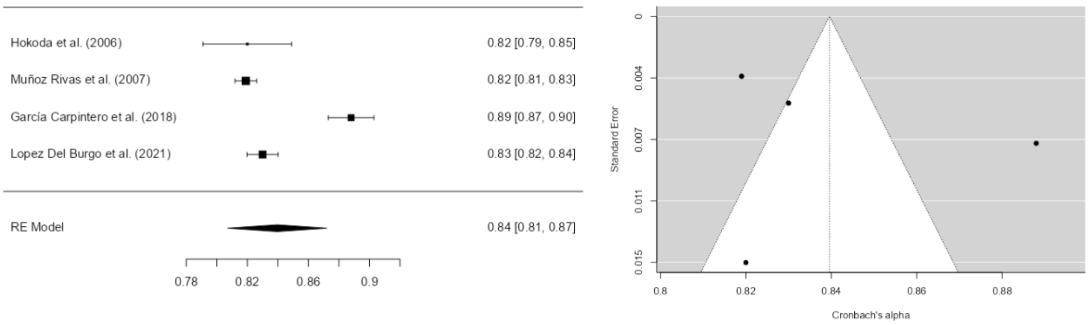

Model 3 (*n* = 3)

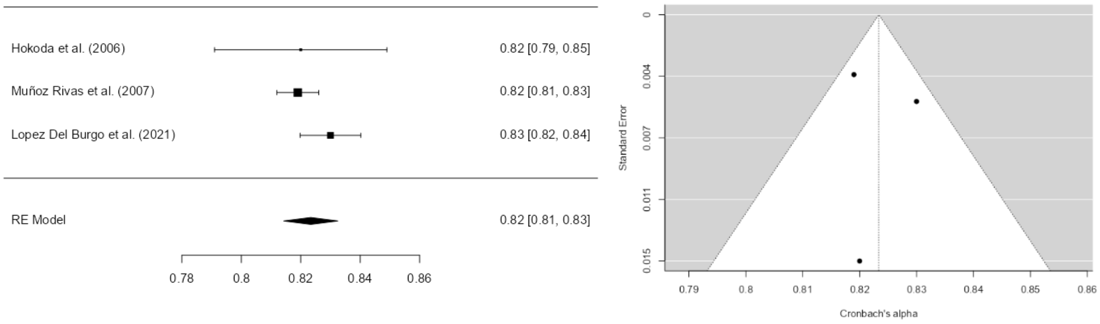

In the dating violence instruments for psychological perpetration in Model 1, the Egger coefficient was 0.472 (p = 0.637) and the I2 was 98.68%. This is the initial model (n = 6). In the second model, studies with an alpha lower than .80 (Fernández Fuertes et al., 2006; Muñoz Rivas et al., 2007) were excluded. Minor differences were found in the coefficients, with Egger = −0.662 (p = 0.508) and I^2 = 85.33%. In the third model, studies with alpha values lower than 0.82 and higher than 0.85 (Soriano Ayala et al., 2021) were excluded. The Egger coefficient was −0.801 (p = 0.423) and the I2 coefficient was 0%. Thus, outliers appear to influence the initial model. However, these results should be interpreted with caution. For further details, refer to Figure S3. Additionally, the most commonly used instruments were CADRI (Conflict in Adolescent Dating Relationship Inventory, Hokoda et al., 2006), CDVI (Cyber Dating Violence Inventory, Morelli et al., 2018), and ADV–YL (Adolescent Dating Violence Questionnaire included in the YourLife Project, Lopez Del Burgo et al., 2021).

Figure S4.

Cronbach's alpha overall sensitivity analysis of the dating violence instruments in sexual perpetration.

Model 1 (*n* = 5)

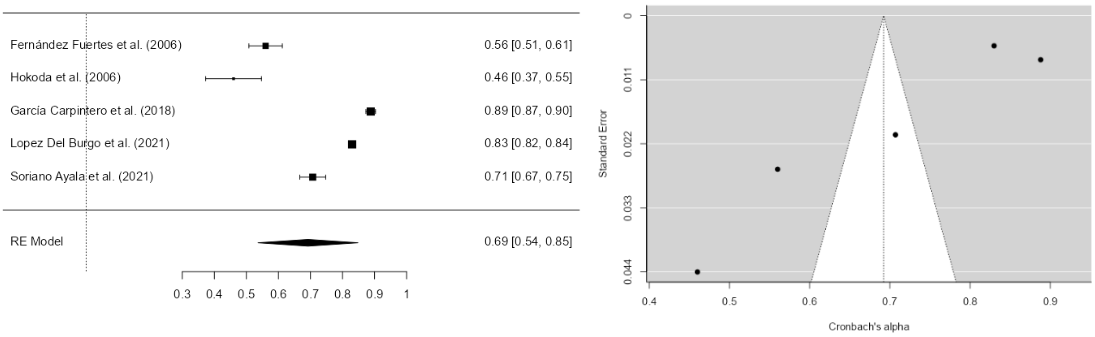

Model 2 (*n* = 3)

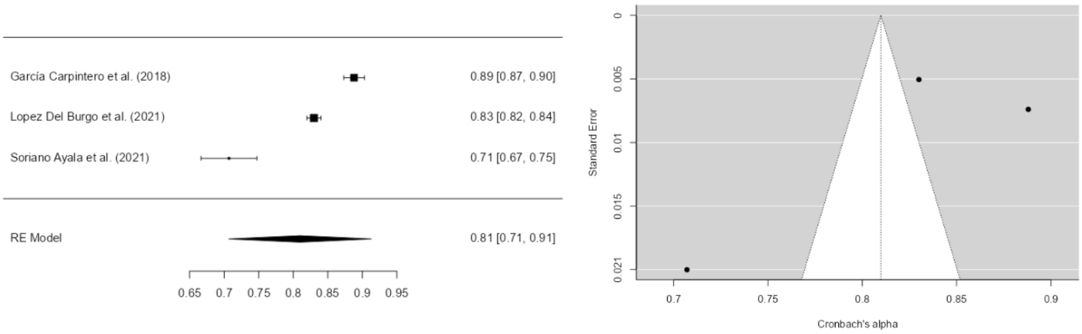

In the dating violence instruments for sexual perpetration in Model 1, the Egger coefficient was -5.861 (p = < 0.001) and the I2 was 99.57%. This is the initial model (n = 5). In the second model, studies with an alpha lower than 0.70 (Fernández Fuertes et al., 2006; Hokoda et al., 2006) were excluded. Minor differences were found in the coefficients, with *Egger* = −1.913 (p= 0.056) and I^2= 99.1%. Thus, outliers appear to influence the initial model. However, these results should be interpreted with caution. For further details, refer to Figure S4. Additionally, the most commonly used instruments were MSDV (Multidimensional Scale Dating Violence, García Carpintero et al., 2018), ADV—YL (Adolescent Dating Violence Questionnaire included in the YourLife Project, Lopez Del Burgo et al., 2021), and TDV-VP (Teen Dating Violence: Victimization and Perpetration Scale, Soriano-Ayala et al., 2021).

Figure S5.

Cronbach's alpha overall sensitivity analysis of the dating violence instruments in psychological victimization.

Model 1 (*n* = 6)

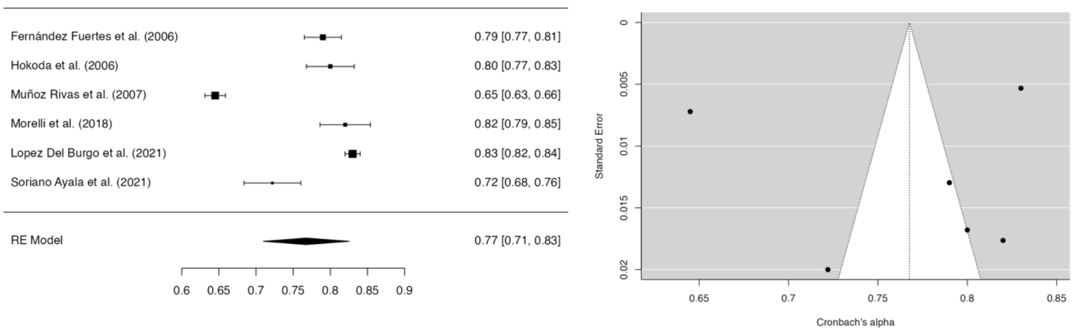

Model 2 (*n* = 3)

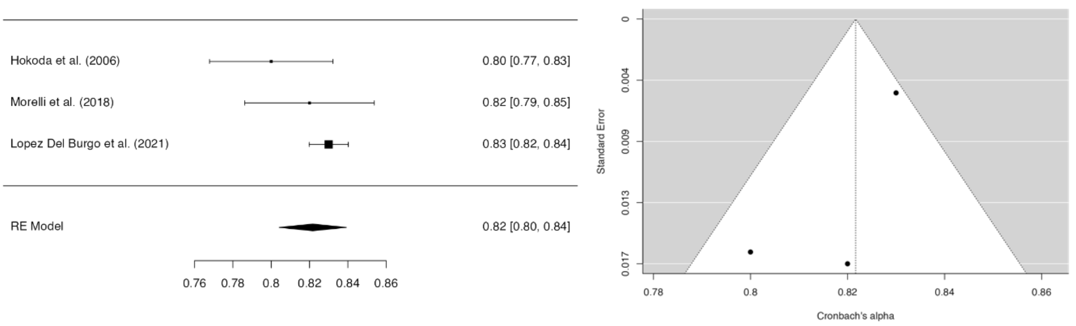

In the dating violence instruments for psychological victimization in Model 1, the Egger coefficient was 0.257 (p = 0.790) and the I2 was 97.96%. This is the initial model (n = 6). In the second model, studies with an alpha lower than .80 (Soriano Ayala et al., 2021; FernándezFuertes et al., 2006; MuñozRivas et al., 2006) were excluded. Minor differences were found in the coefficients, with Egger = −1.543 (p = 0.123) and I2 = 40.3%. Thus, outliers appear to influence the initial model. However, these results should be interpreted with caution. For further details, refer to Figure S5. Additionally, the most commonly used instruments were CADRI (Conflict in Adolescent Dating Relationship Inventory, Hokoda et al., 2006), CDVI (Cyber Dating Violence Inventory, Morelli et al., 2018), and ADV–YL (Adolescent Dating Violence Questionnaire included in the YourLife Project, Lopez Del Burgo et al., 2021).

Figure S6.

Cronbach's alpha overall sensitivity analysis of the dating violence instruments in physical victimization.

Model 1 (*n* = 5)

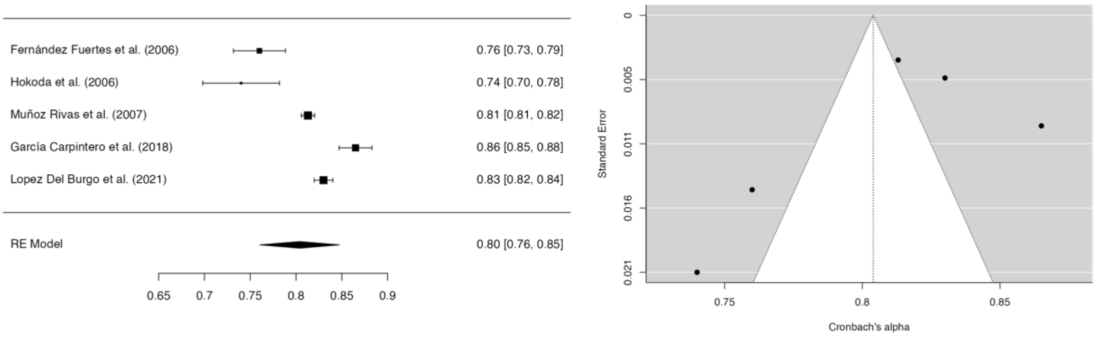

Model 2 (*n* = 3)

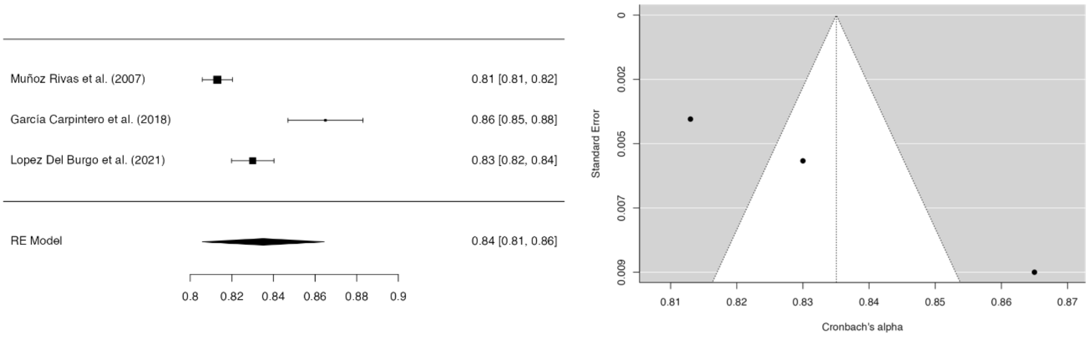

In the dating violence instruments for physical victimization in Model 1, the Egger coefficient was −1.931 (p = 0.053) and the I2 was 97.75%. This is the initial model (n = 5). In the second model, studies with an alpha lower than .80 (Soriano Ayala et al., 2021; Hokoda et al., 2006) were excluded. Minor differences were found in the coefficients, with Egger = 5.449 (p < 0.001) and I2 = 95.38%. Thus, outliers appear to influence the initial model. However, these results should be interpreted with caution. For further details, refer to Figure S6. Additionally, the most commonly used instruments were MSDV (Multidimensional Scale Dating Violence, GarcíaCarpintero et al., 2018), M-CTS (Modified version of the Conflict Tactics Scale, Muñoz Rivas et al., 2007), and ADV–YL (Adolescent Dating Violence Questionnaire included in the YourLife Project, Lopez Del Burgo et al., 2021).

Figure S7.

Cronbach's alpha overall sensitivity analysis of dating violence instruments in sexual victimization.

Model 1 (*n* = 5)

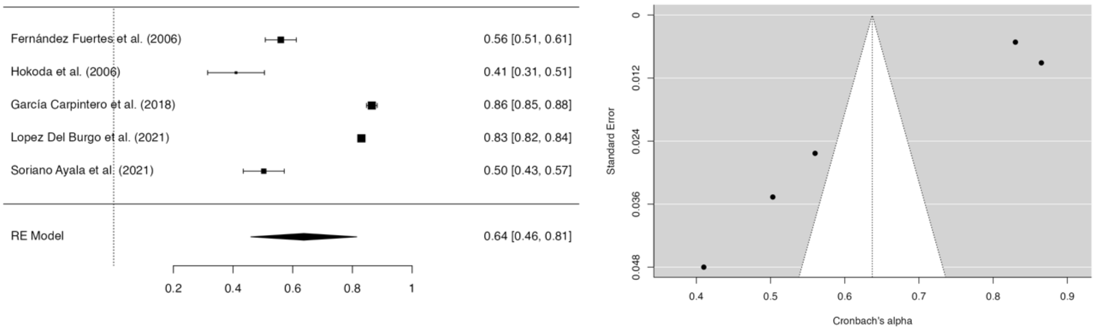

In the dating violence instruments for sexual victimization in Model 1, the Egger coefficient was −6.920 ($p < 0.001$) and the I2 was 99.55%. Thus, outliers appear to influence the initial model. However, these results should be interpreted with caution. For further details, refer to Figure S7. Additionally, the most commonly used instruments were MSDV (Multidimensional Scale Dating Violence, García Carpintero et al., 2018) and ADV–YL (Adolescent Dating Violence Questionnaire included in the YourLife Project, Lopez Del Burgo et al., 2021).

A random-effects meta-analysis was conducted to synthesize the values of the root mean square error of approximation (RMSEA) from 16 eligible studies that measured the model fit for dating violence measurement instruments (Figure S8). The results show an accumulated SRMR estimate of 0.0332 (SE = 0.0100) with a 95% confidence interval (CI) of 0.014–0.053. This value indicates that the model fits well with the observed data. Furthermore, the statistics (I2 = 0%) and (Q = 15.000; $p < 0.001$) indicate moderate heterogeneity among the studies. With a 95% CI in the Egger funnel plot, no publication bias was observed.

A random-effects meta-analysis was also conducted to synthesize the values of the standardized root mean square residual (SRMR) from 4 eligible studies that measured the model fit for dating violence measurement instruments (Figure S8). The results show an accumulated RMSEA estimate of 0.0246 (SE = 0.0238) with a 95% confidence interval (CI) of −0.022 to 0.071. This value suggests a moderately adequate fit in reference to the recommended cutoff of 0.08. Similarly, the statistics (I2 = 0%) and (Q = 0.736; $p < 0.001$) indicate moderate heterogeneity among the studies. With a 95% CI in the Egger funnel plot, no publication bias was observed.

Figure S8.

Forest Plot of all the studies selected for the meta-analysis of RMSEA Y SRMR.

RMSEA (*n* = 16)

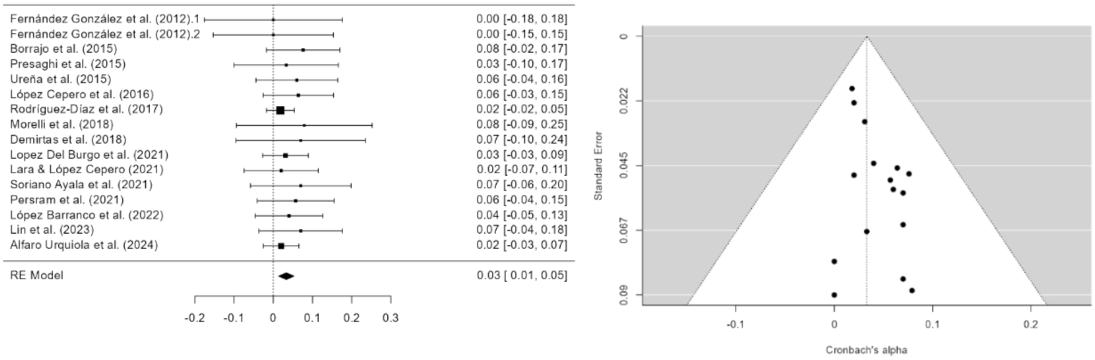

SRMR (*n* = 4)

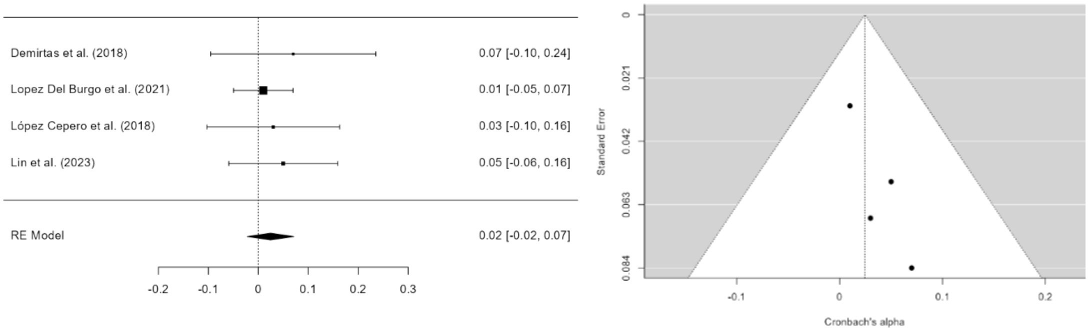

In summary, the reliability and structural validity of instruments measuring dating violence perpetration and victimization were confirmed through a random-effects model and heterogeneity statistics. The overall alpha coefficients for both perpetration and victimization were high, indicating strong reliability across various measures. Specifically, the global alpha was 0.82, and the global omega coefficient was 0.90, both with significant p-values (<0.001), reflecting robust internal consistency.

The alpha coefficients for specific types of violence (psychological, physical, and sexual) also demonstrated substantial reliability, though the coefficients for sexual perpetration and victimization were comparatively lower (0.69 and 0.64, respectively). These lower values suggest that while the instruments are reliable, there is room to improve accuracy in measuring aspects of sexual violence.

The heterogeneity statistics revealed high I^2 values, indicating substantial variability among the studies. Despite this, the strong global reliability coefficients suggest that the instruments consistently measure the intended constructs. The model fit indices (CFI and TLI) were excellent, further supporting the structural validity of these instruments. The CFI was 0.95, and the TLI was 0.96, both with p-values less than 0.001, indicating good fit between the hypothesized model and the observed data. Additionally, the RMSEA and SRMR values were within acceptable ranges, confirming the model's adequacy.

Overall, the findings highlight the effectiveness of these instruments in reliably assessing dating violence perpetration and victimization. The high reliability and validity indices reinforce their utility in both research and clinical

settings. However, the observed variability and slightly lower reliability for sexual violence measures highlight areas for further refinement. These results contribute to the literature by validating these tools and supporting their continued use and development.
